# Characterization of thraustochytrid-specific sterol *O*-acyltransferase: modification of DGAT2-like enzyme to increase the sterol production in *Aurantiochytrium limacinum* mh0186

**DOI:** 10.1128/aem.01001-23

**Published:** 2023-10-24

**Authors:** Yohei Ishibashi, Shohei Sadamitsu, Yoshitomo Fukahori, Yuki Yamamoto, Rin Tanogashira, Takashi Watanabe, Masahiro Hayashi, Makoto Ito, Nozomu Okino

**Affiliations:** 1 Department of Bioscience and Biotechnology, Graduate School of Bioresource and Bioenvironmental Sciences, Kyushu University, Fukuoka, Japan; 2 Kyushu University Future Creators in Science Project (QFC-SP), Fukuoka, Japan; 3 Department of Marine Biology and Environmental Sciences, Faculty of Agriculture, University of Miyazaki, Miyazaki, Japan; University of Illinois Urbana-Champaign, Urbana, Illinois, USA

**Keywords:** sterols, sterol ester, diacylglycerol, thraustochytrid, sterol o-acyltransferase, DGAT

## Abstract

Thraustochytrids are marine microorganisms expected to produce useful lipids. They synthesize polyunsaturated fatty acids and sterols and store them in lipid droplets as a form of triacylglycerol (TG) and sterol ester (SE), respectively. TG is synthesized by diacylglycerol *O*-acyltransferase (DGAT). There are several DGAT2 homologs in *Aurantiochytrium limacinum*. This study indicated that DGAT2C and DGAT2D are SE synthase and TG synthase, respectively, by disrupting their corresponding genes in *A. limacinum* mh0186. DGAT2C is revealed as thraustochytrid-specific acyl-CoA:sterol-*O*-acyltransferase by performing *in vivo* and *in vitro* assays after heterologous expression in *Saccharomyces cerevisiae*. DGAT2C and DGAT2D localized mainly to the endoplasmic reticulum (ER) and the lipid droplet, respectively, and the two of the N-terminal domains unique to DGAT2C were essential for ER localization and SE synthesis in *A. limacinum* mh0186. Interestingly, the study also found that deletion of the first eight transmembrane domains in the unique N-terminal region of DGAT2C increased SE and total sterol productivity when expressed in DGAT2C-deficient *A. limacinum* mh0186. In addition, intermediates such as Δ7-cholesterol (provitamin D3) also accumulated in SE by expression of the N-terminal-truncated DGAT2C, along with cholesterol and Δ7-stigmasterol, a major phytosterol in *A. limacinum*. This study proves that DGAT2C is an ER-localized SE synthase and that its suitable N-terminal deletion can increase sterol production in thraustochytrids.

**IMPORTANCE:**

Since the global market for sterols and vitamin D are grown with a high compound annual growth rate, a sustainable source of these compounds is required to keep up with the increasing demand. Thraustochytrid is a marine oleaginous microorganism that can synthesize several sterols, which are stored as SE in lipid droplets. DGAT2C is an unconventional SE synthase specific to thraustochytrids. Although the primary structure of DGAT2C shows high similarities with that of DGAT, DGAT2C utilizes sterol as an acceptor substrate instead of diacylglycerol. In this study, we examined more detailed enzymatic properties, intracellular localization, and structure-activity relationship of DGAT2C. Furthermore, we successfully developed a method to increase sterol and provitamin D3 productivity of thraustochytrid by more than threefold in the process of elucidating the function of the DGAT2C-specific N-terminal region. Our findings could lead to sustainable sterol and vitamin D production using thraustochytrid.

## INTRODUCTION

Sterols are essential components of the cell membrane of eukaryotes and play an important role in regulating the physicochemical properties, such as membranous strength, fluidity, and permeability, of cell membranes (1). In addition to these fundamental functions, plant sterols (phytosterols) are known to have biological benefits such as anti-inflammatory, anti-oxidant, and anti-cancer activities (2). High blood cholesterol level is a significant risk factor for cardiovascular disease, one of the major causes of death worldwide (3). Importantly, phytosterol can lower blood cholesterol by inhibiting the intestinal absorption of cholesterol (4, 5). Reducing blood cholesterol level by phytosterol is an effective way to reduce the risk of cardiovascular disease. Moreover, highly purified sterol is used as the component of lipid nanoparticles to deliver various therapeutics, such as mRNA, for vaccines against viral infections and cancer (6). Thus, the global market for phytosterols is expected to grow at a compound annual growth rate of 9.6% (5). Oils derived from corn, canola, and wood pulp are the primary sources of phytosterols. However, it has been a concern that terrestrial plants alone may be unable to keep up with the increasing demand for phytosterols because of limited agricultural land and the requirement for a large amount of fresh water. Therefore, alternative sources for sustainable sterol production are required.

Thraustochytrids are eukaryotic marine protists, including the typical genera *Aurantiochytrium*, *Aplanochytrium*, *Schizochytrium*, *Thraustochytrium*, and *Parietichytrium*, which belong to the Stramenopiles, class Labyrinthulomycetes, family Thraustochytriaceae (7 – 9). Thraustochytrids have recently received increasing attention from academic and industry experts because they produce considerable amounts of n-3 polyunsaturated fatty acids (n-3PUFA) such as docosahexaenoic acid (DHA, C22:6 n-3) (10). They accumulate n-3PUFAs and palmitic acid mainly as acyl chains of triacylglycerol (TG) in lipid droplets (LDs) and phosphatidylcholine (PC). In addition to n-3PUFA, several studies have shown that thraustochytrids can synthesize phytosterol (Δ7-stigmasterol) and cholesterol, which are stored as sterol ester (SE) in LDs (11, 12). Because the lipid productivity of thraustochytrids is higher (>50% dry cell weight) than that of terrestrial plants, this marine oleaginous microorganism is expected to be a sustainable source of nutritional lipids, n-3PUFAs, and sterols (13). A key advantage of using thraustochytrid to produce lipids is that they do not require agricultural land, unlike terrestrial plants, and are culturable in seawater, thus minimizing the use of potable fresh water with which scarcity is concerned because of climate change and population growth (5, 14). Since the productivity of sterols by thraustochytrid is insufficient for industrial use, effective methods to improve sterol production are required.

Acyl-CoA:diacylglycerol *O*-acyltransferase (DGAT), which transfers fatty acid from acyl-CoA to diacylglycerol (DG), is a key enzyme for TG synthesis in eukaryotes (15). DGAT is divided into the DGAT1, 2, and 3 families based on their sequence similarities. Lan et al. found that four DGAT2 homologs are present in *Aurantiochytrium*, tentatively named DGAT2A, B, C, and D (16). They showed that DGAT2A, C, and D are TG synthases by heterologous expression in budding yeast. A previous study also indicated that the mRNA expression level of *dgat2d* is higher than that of *dgat2a* and *dgat2c*, suggesting that *dgat2d* would be a major TG synthase in *A. limacinum* (16).

In this study, we examined the endogenous function of DGAT2 homologs in *A. limacinum* mh0186 and revealed that DGAT2C synthesizes SE by using acyl-CoA and sterols as substrates, and suitable deletion of N-terminal transmembrane regions of DGAT2C increased the sterol production, including phytosterols in *Aurantiochytrium limacinum* mh0186. Furthermore, expression of the N-terminal-deletion mutant in *A. limacinum* mh0186 produced Δ7-cholesterol-ester, which is rarely detected in wild-type (WT) *A. limacinum*.

## MATERIALS AND METHODS

### Strains and culture

*A. limacinum* mh0186 (17), isolated from seawater of the Yaeyama Islands in Okinawa, Japan, was grown in GY medium (3% glucose and 1% yeast extract in 1.75% artificial seawater) at 25°C with rotation at 150 rpm for the period indicated. Potato dextrose agar (PDA) plates (50% potato dextrose, 1.75% artificial sea water, 2% agar) containing appropriate antibiotics were used to select the *dgat2a* knockout (KO), *dgat2c* KO, *dgat2d* KO, *dgat2c* KO/overexpression (OE), *dgat2c* OE, and N-terminal transmembrane regions deleted (ΔTM) *dgat2c*-expressing strains. The growth (biomass) of *A. limacinum* WT and transfected strains were monitored by measuring optical density at 600 nm (OD 600). The glucose concentration of the medium was measured by the Glucose CII test (FUJIFILM Wako Pure Chemical Co., Japan).

### Materials

1,2-Diundecanoyl-*sn*-glycero-3-phosphocholine [PC22:0 (C11:0/C11:0)], 1,2-didocosahexaenoyl-*sn*-glycero-3-phosphocholine [PC44:12 (C22:6/C22:6)], docosahexaenoyl coenzyme A (C22:6-CoA), and 16:0 cholesteryl-d7 ester (d7-C16:0-CE) were purchased from Avanti Polar Lipids (AL, USA). TG36:0 (12:0/12:0/12:0) (Trilaurin) was purchased from Tokyo Chemical Industry (Tokyo, Japan). Antibodies against anti-DYKDDDDK tag (#2368) and anti-rabbit IgG horseradish peroxidase (HRP) were obtained from Cell Signaling Technology (MA, USA). SEALIFE, used as artificial seawater, was purchased from Nihonkaisui Co. (Tokyo, Japan). Thin-layer chromatography (TLC) plate was purchased from Merck Millipore (MA, USA).

### Generation of *dgat2a*-, *dgat2c*-, or *dgat2d*-disrupted mutants

The genetic information of *dgat2a*, *dgat2c*, and *dgat2d* was obtained using the draft genome database provided by Joint Genome Institute. Genes encoding *dgat2a* (GenBank accession number: MT762143), *dgat2c* (GenBank accession number: MF926506.1), and *dgat2d* (GenBank accession number: MF926507.1) were disrupted by homologous recombination using a targeting construct (Fig. S1A through C) by the method described in reference (18). 5′ and 3′ homology arms of *dgat2a*, *dgat2c*, or *dgat2d* were amplified using the genomic DNA of *A. limacinum* as a template with primers described in Table S1. The antibiotic-resistant gene-expressing construct was connected with 5′ and 3′ homology arms by fusion PCR mediated with PrimeSTAR GXL DNA Polymerase (Takara Bio Inc., Shiga, Japan). The knockout construct was introduced into *A. limacinum* by electroporation with a Gene Pulser Xcell (Bio-Rad, Inc., CA, USA) (750 V, 25 µF, 200 Ω). After being pulsed twice, 800 µL of fresh GY medium was added. Cells were cultured at 25°C for 1 day and then transferred to a PDA plate containing 0.5 mg/mL of G418 or 1 mg/mL of hygromycin B (Wako Chemicals Inc., Osaka, Japan). The *dgat2* KO strains were selected and confirmed by PCR (Fig. S1D through F).

### Lipid analysis

For TLC analysis, cells were cultured in GY medium at 25°C with shaking at 150 rpm for 3 or 8 days. Cells were collected by centrifugation (5,000 × *g*, 3 min) then dried by lyophilization. Total lipid was extracted from 5 mg of the dried cell by adding 500 µL of chloroform/methanol (2:1, vol/vol) with sonication. After incubation at 25℃ for 10 min, samples were centrifuged at 17,500 × *g* for 15 min, then 10 µL of supernatant was loaded onto TLC, which was developed with hexane/diethyl ether/acetic acid (50/50/1 or 80/20/1, vol/vol/vol) for 10 min. Neutral lipids were visualized by spraying copper sulfate solution with heating at 120°C. For liquid chromatography-electrospray ionization-tandem mass spectrometry (LC-ESI MS/MS) analysis, cells were cultured in GY medium at 25°C with shaking at 150 rpm for an appropriate period, then 500 µL of culture fluid was harvested. Cells were collected by centrifugation (5,000 × g, 3 min), then the cell pellet was resuspended in 150-µL distilled water, then crushed at 3,000 rpm for 60 s using a bead beater (μT-12; TAITEC, Saitama, Japan) with glass beads (diameter: 0.6 mm, AS ONE Corp., Osaka, Japan) and kept on ice for 60 s. This procedure was repeated three times to prepare the cell lysate. Cellular lipids were extracted from 40 µL of cell lysate by adding 160 µL of chloroform/methanol (2:1, vol/vol) containing 10-µM PC22:0 (C11:0/C11:0), 20-µM DG24:0 (C12:0/C12:0), 10-µM d7-C16:0-CE, and 20-µM TG36:0 (C12:0/C12:0/C12:0) as internal standards. After incubation at 37°C for 30 min, the mixture was centrifuged at 11,000 × *g* for 3 min. Forty microliters of the organic phase was transferred to autoinjector vials containing 560 µL of 2-propanol, and then the cellular lipids were measured using LC-ESI MS/MS (3200 QTRAP; SCIEX, MA, USA). A binary solvent gradient with a 200-µL/min flow rate was used to separate phospholipids and neutral lipids by reverse-phase chromatography using an InertSustain C18 column (2.1 × 150 mm, 5 µm; GL Sciences, Japan) as described in references (19, 20). PC, DG, TG, and SE containing palmitic acid (C16:0) and DHA (C22:6) were detected using multiple reaction monitoring (MRM) as described in Table S2. For the analysis of TG and ergosterol ester (EE) of *dgat2*c- and *dgat2d*-expressing yeasts, palmitoleic acid- (C16:1) and oleic acid (C18:1)-containing molecular species were added to the MRM (Table S2). The signal values of each lipid were normalized with their corresponding internal standards. To measure the total sterol in *A. limacinum,* methanolysis using an acid catalyst [methanol containing 8% (wt/vol) HCl] was performed to release the esterified fatty acid of SE to generate a free form of sterols, as described in reference (21). The amount of free-form sterols was measured using liquid chromatography-atmospheric pressure chemical ionization (LC-APCI) MS/MS (3200 QTRAP) after separating sterols by a binary solvent gradient with an 800-µL/min flow rate using an InertSustain C18 column (2.1 × 150 mm) started with 0% solvent B (methanol with 0.1% acetic acid) in solvent A (methanol/water = 96:4 containing 0.1% acetic acid) (22). The gradient reached 90% B over 4 min, then 100% B over 8 min, and was maintained for 2 min. The gradient was returned to the starting conditions, and the column was equilibrated for 4 min before the next run.

### Assay of the *in vitro* TG synthase activity

WT and *dgat2d* KO strain were cultured until the glucose concentration of the medium reached approximately 0.5%. Two milliliters of the culture was collected and resuspended in 300 µL of 90-mM HEPES, pH 7.4, containing 0.02% Tween 20, and protease inhibitor cocktail (cOmplete Mini, Merck Millipore) then crushed by a beads beater. The amount of protein in the lysate was determined using a bicinchoninic acid protein assay (Thermo Fisher Scientific, USA). The cell lysate equivalent to 30 µg of protein was incubated in 60 µL of 15-mM Tris-HCl buffer, pH 7.5, containing 15 µM of [^14^C]palmitoyl-CoA (0.1 mCi/mL, American Radiolabeled Chemicals Inc., USA), 25 mM of sucrose, 15 mM of KCl, 15 mM of MgCl_2_, and 125 µg/mL of fatty acid-free bovine serum albumin (BSA) at 30°C for 10 min. The reaction was stopped by adding 300 µL of chloroform/methanol (2/1, vol/vol). The organic phase was evaporated under nitrogen gas, then resuspended in 10 µL of chloroform/methanol (2/1, vol/vol), then loaded onto TLC that was developed with hexane/diethyl ether/acetic acid (50/50/1, vol/vol/vol). The radioactivity was measured using an FLA 5100 Bio-imaging analyzer (GE Healthcare).

### Generation of *dgat2*c-overexpressing mutants

To prepare the construct expressing FLAG-tagged *dgat2*c, PCR was performed using genomic DNA derived from *A. limacinum* mh0186 as a template and the primer described in Table S1. Inverse PCR using pEF-Neor/Ubi-mCherry as template DNA was performed to eliminate mCherry, which was located between the ubiquitin promoter and ubiquitin terminator derived from *Thraustochytrium aureum* to express the gene of interest in thraustochytrids, using primer set A.L_vector_Rv and A.L_vector_Fw (23) (Table S1). C-terminal FLAG-tagged *dgat2*c was inserted between the ubiquitin promoter and ubiquitin terminator of pEF-Neor/Ubi-mCherry instead of mCherry using an In-Fusion HD cloning kit (Takara Bio Inc.). The plasmid was used as a template to generate *dgat2c* mutants with truncated N-terminal predicted transmembrane regions (see Fig. 5A) amplified by PCR using primers listed in Table S1. To prepare the construct expressing N- or C-terminal green fluorescent protein (GFP)-fused DGAT2C or DGAT2D, PCR was performed using the primer set described in Table S1. The expression vector of enhanced GFP (EGFP) for thraustochytrids, pEF-Neor/Ubi-EGFP (pENUG) (23), was linearized by PCR using primers described in Table S1. The open reading frames (ORFs) of *dgat2c* or *dgat2d* were inserted between *egfp* and ubiquitin terminator of pENUG to make N-terminal GFP-fused protein and between ubiquitin promoter and *egfp* of pENUG to make C-terminal GFP-fused protein using an In-Fusion HD cloning kit, respectively. The linearized expression construct was prepared by PCR using plasmid as a template, then introduced into *A. limacinum* mh0186 by electroporation using Gene Pulser Xcell (750 V, 25 µF, 200 Ω). The transformants that grew on the PDA plate containing 0.5-mg/mL G418 were subjected to PCR screening.

### Expression of *dgat2c* and *dgat2d* in *Saccharomyces cerevisiae*

The ORFs of *dgat2c* and *dgat2d* were amplified by PCR using genomic DNA of *A. limacinum* mh0186 and inserted into the MCS of pYES2/CT (Invitrogen, MA, USA). FLAG-tag was preadded to pYES2/CT, and *dgat2*c and *dgat2d* were expressed as C-terminal FLAG-tagged proteins. The expression plasmid vector of *dgat2c* was used as a PCR template to generate H642A and H644A mutant amplified using mutant primers listed in Table S1. The expression vectors were introduced into *S. cerevisiae* INVSc1 (Invitrogen) using the previously described method (24). The transformants were selected by plating on synthetic agar plates lacking uracil (SC-ura). *S. cerevisiae* transformants harboring *dgat2c* and *dgat2d* were cultured in SC-ura medium containing 2% glucose at 30°C for 1 day and cultured for an additional day in SC-ura medium containing 2% galactose. Next, 500 µL of culture fluid was centrifuged at 1,500 × *g* for 5 min to collect the cells and was used for the lipid analysis.

### Quantitative real-time PCR

Total RNA was extracted from *A. limacinum* mh0186 after 5 days of culture using Sepasol-RNA I Super G (Nacalai Tesque) and an SV Total RNA Isolation System (Promega). cDNA was synthesized using a PrimeScript RT Reagent Kit with a gDNA Eraser (Perfect Real Time) (Takara Bio Inc.). Real-time PCR was performed using an Mx3000P qPCR System (Agilent Technologies) with SYBR Premix Ex Taq II (Tli RNaseH Plus) (Takara Bio Inc.) by using cDNA as a template. The numbers of copies of *dgat2a*, *dgat2c*, and *dgat2d* were determined using a plasmid containing each gene as a standard. Oligonucleotide primer sets for real-time PCR are shown in Table S1.

### Western blotting analysis

To detect FLAG-tagged proteins in yeast and *A. limacinum*, 1 mL and 200 µL of culture fluid were used, respectively. Cell pellets were suspended in PBS containing protease inhibitor cocktail and crushed by a beads beater with glass beads as described above. The amount of protein in the lysate was determined using a bicinchoninic acid protein assay. Equal amounts of proteins were subjected to 12.5% SDS-PAGE, followed by blotting onto a PVDF membrane using a Trans-Blot SD semi-dry transfer cell (Bio-Rad, Inc.). The membrane was blocked in Tris-buffered saline-Tween 20 (TBS-T) containing 5% BSA, then incubated with antibodies against DYKDDDDK tag (1:5,000) for over 12 h at 4°C. The membrane was washed with TBS-T and then incubated with HRP-conjugated anti-rabbit IgG antibody (1:10,000) (Cell Signaling Technology). The membrane was washed with TBS-T, and the protein was detected by chemiluminescence using Luminata Forte Western HRP substrate (Merck Millipore) and visualized with the use of a Cooled CCD Camera System Ez-Capture II (ATTO).

### Assay of the *in vitro* SE synthase activity of DGAT2C

*S. cerevisiae* transformants harboring *dgat2c* or mock vector were cultured in 20 mL of SC-ura medium containing 2% glucose in a 50-mL flask at 30°C with shaking at 150 rpm for 1 day, and then cultured for an additional day in 40-mL SC-ura medium containing 2% galactose in a 100-mL flask. Twenty milliliters of the culture was centrifuged at 1,500 × *g* for 5 min; then, cell pellets were dissolved in 300 µL of PBS containing protease inhibitor cocktail and then crushed by a beads beater. The cell lysate was transferred to a 1.5-mL tube and centrifuged at 1,000 × *g* for 3 min. The supernatant was transferred to an ultracentrifuge tube, then centrifuged at 100,000 × *g* for 1 h at 4°C to obtain the microsomal fraction. After removing the supernatant, the pellet was resuspended with sterile distilled water, then the protein concentration of the microsome was measured with a bicinchoninic acid protein assay. One hundred pmol of ergosterol was mixed with 300 nmol of sodium cholate. The mixture was sonicated for 30 s, and then the solvent was evaporated by a speed vac concentrator. The dried substrate was dissolved in 40 µL of 15 mM Tris-HCl buffer, pH 7.5, containing 33.3 µM of C22:6-CoA, 25 µM of sucrose, 15 µM of KCl, 15 µM of MgCl_2_, and 125 µg/mL of fatty acid-free BSA with sonication (30 s). Finally, a microsomal fraction equivalent to 40 µg of protein was added to the substrate mixture, then incubated at 37℃ with shaking at 1,500 rpm for 8 h. To determine the acyl donor of DGAT2C, 2 nmol of 1,2-didocosahexaenoyl-*sn*-glycero-3-phosphocholine (C22:6 PC, Avanti Polar Lipids) or docosahexaenoic acid (C22:6 free, Avanti Polar Lipids) was added to the reaction mixture instead of C22:6-CoA. The reaction was stopped by adding 400 µL of chloroform/methanol (2/1, vol/vol), and the sample was centrifuged (11,000 × *g* for 3 min). One hundred microliters of the organic phase was transferred to autoinjector vials containing 500 µL of 2-propanol, and then the product, C22:6-erogsterol ester (C22:6-EE), was measured using LC-ESI MS/MS.

### Fluorescence microscopy

The N- or C-terminal GFP-fused DGAT2C or DGAT2D was observed under a fluorescence microscope, DMi8 with Leica Application Suite X (LAS X), equipped with an objective lens of ×100 (numerical aperture 1.40) and a DFC3000G camera (Leica Microsystems, Wetzlar, Germany). LDs were stained by high-content screening LipidTOX Red neutral lipid stain, and nuclei were stained by Hoechst 33342 (Thermo Fisher Scientific).

### Statistical analysis

All statistical analyses were performed using unpaired two-tailed Student *t*-tests, one-way or two-way analysis of variance with Tukey, and all data are expressed as means and standard deviations from at least three separate experiments. Statistical significance is indicated as follows: *P < 0.05, **P < 0.01, and ***P < 0.001.

## RESULTS

### Genetic analysis of *dgat2a*, *dgat2c*, and *dgat2d* in *A. limacinum* mh0186

**Fig 1:**
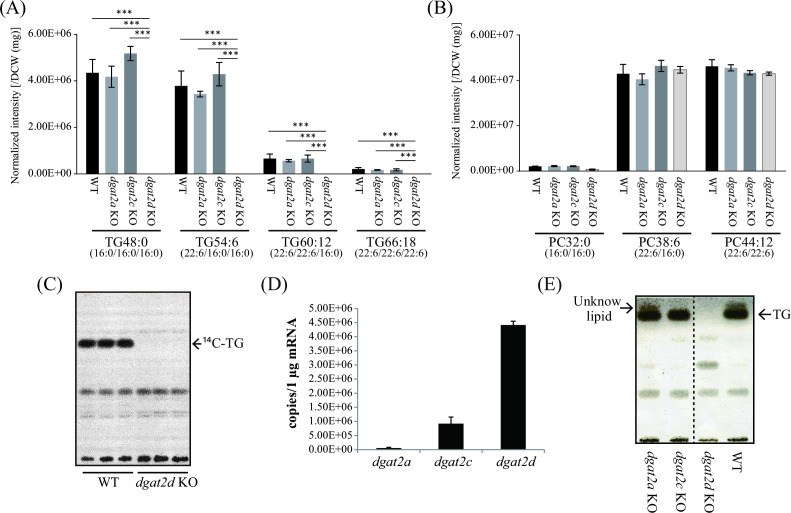
Lipid profile of *dgat2a*, *dgat2*c, and *dgat2d* KO strains. Quantification of each molecular species of TG (**A**) and PC (**B**) of WT, *dgat2a*, *dgat2c*, and *dgat2d* KO strains harvested from 3-day cultures. The results of panels **A **and **B **were obtained by LC-ESI MS/MS analysis (MRM mode). The peak intensity of each lipid was normalized with dry cell weight (DCW). Data represent means ± SDs of three separate experiments. Statistical significance is indicated as follows: ***P < 0.001. (**C**) *In vitro* TG synthase activity of WT and *dgat2d* KO strain. TG synthase activity was measured using ^14^C-labeled palmitoyl-CoA as a donor substrate, and cell lysate as an enzyme and acceptor substrate (DG) source, as described in Materials and Methods. (**D**) mRNA expression levels of *dgat2a*, *dgat2c*, and *dgat2d*. A standard curve to determine the copy numbers of each gene was generated from various concentrations of plasmid containing the DNA sequence of *dgat2a*, *dgat2c*, and *dgat2d*, respectively. (**E**) TLC analysis showing the neutral lipid profiles of *dgat2a*, *dgat2c*, *dgat2d* KO strains and WT. Total lipids were extracted from cells cultured for 8 days. Lipid extracts were applied to a TLC plate that was developed with hexane/diethyl ether/acetic acid = 50/50/1 (vol/vol/vol). Neutral lipids were visualized by spraying copper sulfate solution. Noncontiguous portions of the same TLC are indicated by dashed lines.

To elucidate the function of DGAT2 homologs in more detail, *dgat2a*, *dgat2c*, and *dgat2d* were disrupted in *A. limacinum* mh0186 (designated *dgat2a* KO, *dgat2c* KO, and *dgat2d* KO, respectively) (Fig. S1). LC-ESI MS/MS analysis showed that amounts of all molecular species of TG were dramatically reduced in the *dgat2d* KO (Fig. 1A). PC of *dgat2a*, *dgat2c*, and *dgat2d* KO was similar to that in the WT strain (Fig. 1B). In agreement with the reduction of TG, DG, which is a precursor for the synthesis of TG, was increased in *dgat2d* KO (Fig. S2A and B). In contrast, all molecular species of TG were not decreased in the *dgat2a* KO and *dgat2c* KO stains in comparison to those in the WT (Fig. 1A). Although DGAT2A has been reported to have TG synthetic activity (16), it seems that DGAT2A contributes little to the endogenous TG synthesis in *A. limacinum*. Importantly, the *in vitro* TG synthetic activity of cell lysate was markedly decreased in *dgat2d* KO, despite the presence of other *dgat2* homologs in this strain (Fig. 1C). These results indicate that DGAT2D is a major TG synthase that mainly contributes to the TG synthesis in *A. limacinum*, and other DGAT2 homologs are unable to compensate for the loss of DGAT2D function. The quantitative PCR showed that the mRNA expression level of *dgat2d* is significantly higher than that of other *dgat2* homologs (Fig. 1D), which is consistent with the result described in reference (16). This suggests that *dgat2* homologs with low expression levels are unlikely to reach sufficient transcript levels to compensate for the loss of *dgat2d*. The *dgat2d* KO strain showed a marked decrease in turbidity measured at OD600 and a delay in the consumption of glucose, the main carbon source in the culture medium, indicating that disruption of *dgat2d* causes the severe growth defect in *A. limacinum* (Fig. S3A and B). On the other hand, the growth of *dgat2a* KO and *dgat2c* KO strains was almost identical to that of the WT (Fig. S3C and D), suggesting that TG synthesis could be important for *A. limacinum* for normal growth. Since TG synthetic activity was almost completely abolished by *dgat2d* KO (Fig. 1C), we consider that the reduction of TG was due to the loss of TG synthesis mediated by DGAT2D, rather than the growth defect in the *dgat2d* KO strain.

**Fig 2:**
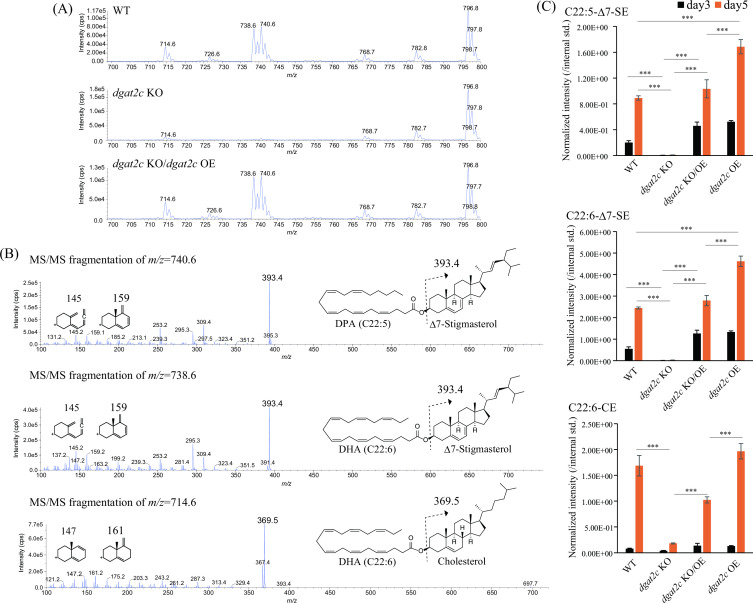
Structural analysis of lipids lost in the *dgat2c* KO strain of *A. limacinum* mh0186. (**A**) Positive ion MS spectra of SE that were lost in the *dgat2c* KO strain. The spectrum shows that *m*/*z* = 740.6 and 738.6 were ablated in the *dgat2c* KO strain. In comparison with that of the WT, the intensity of *m*/*z* = 714.6 decreased in the *dgat2c* KO strain. The intensities of these spectra were recovered by the reintroduction of *dgat2c* into the *dgat2c* KO strain. (**B**) MS/MS fragmentation spectra of *m*/*z* = 740.6, 738.6, and 714.6. Structure-specific fragment ions are generated in MS/MS, depending on the number of the double bond in ring B of sterol moieties of SE. According to their MS/MS fragmentation pattern, *m*/*z* = 740.6, 738.6, and 714.6 were identified as docosapentaenoic acid (DPA, C22:5)-Δ7-stigmasterol ester (C22:5-Δ7-SE), C22:6-Δ7-stigmasterol ester (C22:6-Δ7-SE), and C22:6-cholesterol ester (C22:6-CE), respectively. (**C**) Quantification of SE of the WT, *dgat2c* KO, *dgat2c* KO/*dgat2c* OE, and *dgat2c* OE strains. The cellular C22:5-Δ7-SE, C22:6-Δ7-SE, and C22:6-CE levels of each strain were measured using LC-ESI MS/MS (MRM mode) at the indicated time points. Data represent means ± SDs of three separate experiments. The peak intensity of each lipid was normalized with that of the internal standard. Statistical significance is indicated as follows: ***P < 0.001.

In agreement with LC-ESI MS/MS result, TLC analysis revealed that the band corresponding to TG disappeared in *dgat2d* KO, while the band remained unchanged in the WT, *dgat2a* KO, and *dgat2*c KO (Fig. 1E). We found that an unknown lipid whose *R*_f_ is faster than TG on the TLC detected in the WT disappeared in the *dgat2c* KO (Fig. 1E). To identify this unknown lipid, MS analysis of lipids from the WT, *dgat2c* KO, and *dgat2c* KO/*dgat2c* OE, which is the strain that *dgat2*c was reintroduced in the *dgat2*c KO, were performed. Consequently, the intensity of the spectra corresponding to *m*/*z* = 714.6 was significantly reduced, and that corresponding to *m*/*z* = 738.6 and 740.6 lost in the *dgat2*c KO compared to the WT and *dgat2c* KO/*dgat2c* OE (Fig. 2A). Tandem mass spectrometry (MS/MS) analysis revealed that the *m*/*z* = 740.6 and 738.6 spectra were derived from Δ7-stigmasterol-ester (Δ7-SE) containing docosapentaenoic acid (C22:5-Δ7-SE) and DHA (C22:6-Δ7-SE), respectively, and that of *m*/*z* = 714.6 was from cholesterol ester (CE) containing DHA (C22:6-CE), because fragment ion *m*/*z* = 393.4 derived from precursor ions *m*/*z* = 740.6 and 738.6 was assigned to Δ7-stigmasterol (25), and *m*/*z* = 369.5 derived from precursor ion *m*/*z* = 714.6 was assigned to cholesterol in the MS/MS analysis (Fig. 2B). These results were supported by detecting the fragment ions *m*/*z* = 145 and *m*/*z* = 159 that were derived from double bonds at Δ5 and Δ7 of the B-ring in Δ7-stigmasterol (Fig. 2B), and the fragment ions *m*/*z* = 147 and *m*/*z* = 161 that were derived from a single double bond at Δ5 of the B-ring in cholesterol (Fig. 2B) (26). The amounts of polyunsaturated fatty acid PUFA)-containing SE drastically decreased in the *dgat2c* KO in comparison to the WT and *dgat2c* KO/OE (Fig. 2C). In contrast to DHA, palmitic acid (C16:0) is rarely incorporated into SE in *A. limacinum* mh0186 (Fig. S4). These results indicate that DGAT2C is involved in PUFA-containing SE synthesis in *A. limacinum* mh0186. Subsequently, the *dgat2c* was overexpressed in the WT (*dgat2c* OE strain) to further understand the function of DGAT2C. The amounts of SE, particularly C22:5-Δ7-SE and C22:6-Δ7-SE, increased in the *dgat2c* OE cultured for 5 days (Fig. 2C).

### *In vivo* and *in vitro* assays of DGAT2C after heterologous expression in budding yeast

**Fig 3:**
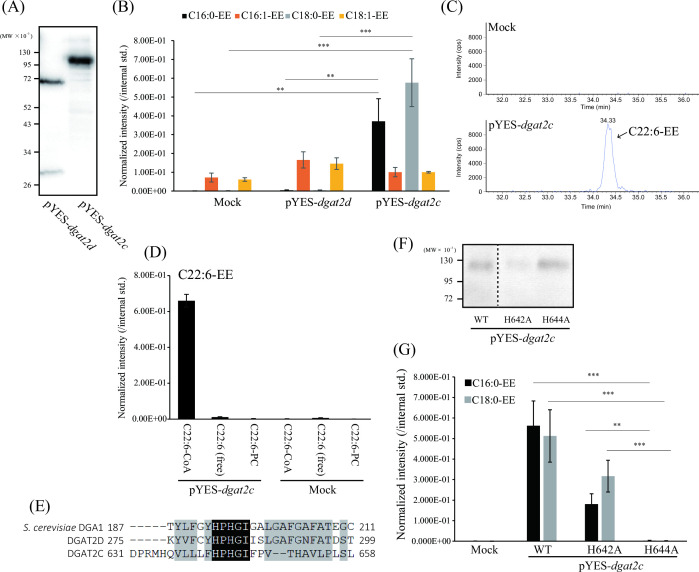
*In vivo* and *in vitro* assays of DGAT2C after heterologous expression in budding yeast. (**A**) Western blotting showing the expression of DGAT2D and DGAT2C in *S. cerevisiae*. The C-terminal FLAG-tagged DGAT2D and DGAT2C were expressed in *S. cerevisiae* using the pYES vector. After induction of protein expression by galactose, cells were collected and crushed by a bead beater. Cell lysates were subjected to Western blotting using an antibody against FLAG-tag. (**B**) Quantification of cellular ergosterol-ester (EE) levels of mock, *dgat2d*, and *dgat2c*-expressing *S. cerevisiae*. EE containing C16:0, C16:1, C18:0, and C18:1 of each strain was measured by LC-ESI MS/MS (MRM mode). (**C**) *In vitro* SE synthetic activity of DGAT2C expressed in *S. cerevisiae*. The microsomal fractions equivalent to 40 µg of protein derived from the mock and *dgat2c*-expressing strains were mixed with ergosterol and C22:6-CoA, as described in the Materials and Methods. After incubation at 37°C for 8 h, an appropriate amount of chloroform/methanol = 2/1 (vol/vol) was added to the reaction solutions. The organic phase containing SE was applied to LC-ESI MS/MS (MRM mode) analysis to detect C22:6-EE. (**D**) Identification of the acyl donor substrate for the esterified fatty acid of SE synthesis by DGAT2C. The same amount of C22:6-CoA, free form of C22:6 [C22:6 (free)], or PC comprising C22:6 (C22:6-PC) was incubated with microsomal fractions of the mock and *dgat2c-*expressing yeast to reveal the acyl donor of DGAT2C. (**E**) Multiple alignments of amino acid sequences around the HPHG motif, which is conserved in the DGAT2 family enzyme, of DGA1 (TG synthase of *S. cerevisiae*), DGAT2D, and DGAT2C. (**F**) Western blot showing the expression levels of the WT, H642A, and H644A mutants of DGAT2C in *S. cerevisiae*. Noncontiguous portions of the same membrane are indicated by dashed lines. (**G**) Quantification of EE levels of the mock, *dgat2c* (WT)-, *dgat2c* (H642A)-, and *dgat2c* (H644A)-expressing *S. cerevisiae*. Data represent means ± SDs of three separate experiments. The peak intensity of each lipid was normalized with that of the internal standard. Statistical significance is indicated as follows: **P < 0.01, and ***P < 0.001.

Rau et al. reported that dysfunction of AlASATb, which was found as a DGAT2 homolog, significantly reduced the intracellular SE level in *A. limacinum* SR21 and suggested that AlASATb was an acyl-CoA:sterol-*O*-acyltransferase (ASAT) (27). To further understand the functions of DGAT2 homologs, the C-terminal FLAG-tagged *dgat2c* and *dgat2d* were expressed in *S. cerevisiae*, and their expressions were examined by Western blotting using an anti-FLAG-tag antibody. DGAT2D (60.7 kDa) and DGAT2C (99.9 kDa) were detected with the expected molecular weight, indicating that these genes are properly expressed in *S. cerevisiae* (Fig. 3A). The amount of TG increased in the *dgat2d*-expressing strain (pYES-*dgat2d*), while that of the *dgat2c*-expressing strain (pYES-*dgat2c*) was almost the same as that of the empty vector expressing strain of *S. cerevisiae* (mock) (Fig. S5). In the mock strain, C16:1-EE and C18:1-EE, but not C16:0-EE and C18:0-EE, were detected (Fig. 3B) due to the specificity of yeast SE synthase (Are1/2) and endogenous sterol pool in *S. cerevisiae*; i.e., Are1/2 prefers monounsaturated fatty acids such as palmitoleic acid (C16:1) and oleic acid (C18:1), and the major sterol pool in *S. cerevisiae* is ergosterol (28). In contrast to the mock, C16:0-EE and C18:0-EE significantly increased in pYES-*dgat2c*, but not pYES-*dgat2d* (Fig. 3B), indicating that DGAT2C has an SE synthase activity with different specificity from Are1/2 of *S. cerevisiae*. Next, the SE synthase activity was examined *in vitro* using the microsomal fraction of pYES-*dgat2c* as an enzyme source. C22:6-EE was detected when the microsomal fraction of pYES-*dgat2c*, but not the mock, was incubated with C22:6-CoA (Fig. 3C and D). However, a free form of DHA [C22:6 (free)] or DHA-containing PC (C22:6-PC) was not a donor substrate for DGAT2C (Fig. 3D). Taken together, *in vivo* and *in vitro* experiments using *S. cerevisiae* clarified that DGAT2C is an SE synthase that uses acyl-CoA as a donor substrate, and, thus, the systematic name of DGAT2C should be ASAT, although the primary structure is more homologous to DGAT2 than to known ASATs.

### Identification of catalytic residue of DGAT2C

The HPHG motif conserved in all DGAT2 family members is required for the enzymatic activity of DGAT2; thus, this motif is considered a part of the active site (29). This motif is conserved in DGAT2D and DGAT2C (Fig. 3E). Because DGAT2C is the first DGAT2 that uses sterols as acceptor substrates, whether this motif is essential for enzyme activity, such as conventional DGAT2, is unclear. Since histidine residue is known to be involved in catalysis in DGAT1 (29), the first and third residues in the HPHG motif of DGAT2C were replaced with alanine (H642A and H644A), respectively, and the activity of each mutant was compared with the WT enzyme. Although the protein expression level of the H644A mutant enzyme (H644A) was almost the same as that of DGAT2C (WT) in *S. cerevisiae* (Fig. 3F), C16:0-EE and C18:0-EE, which were synthesized by DGAT2C (WT), were not detected in the H644A-expressing strain, similarly to the mock transfectant (Fig. 3G). On the other hand, C16:0-EE and C18:0-EE were synthesized in an H642A-expressing strain despite lower protein expression level compared to WT and H644A (Fig. 3F and G), suggesting that the first residue (histidine) of the HPHG is not involved in the catalyst of DGAT2C but might contribute to the protein stability of DGAT2C. Taken together, our results indicate that the third residue (histidine) of the HPHG motif is essential for the ASAT activity of DGAT2C similar to the conventional DGAT2.

### Different intracellular localization of DGAT2C and DGAT2D

As described above, two DGAT2 homologs, DGAT2C and DGAT2D, possess completely different substrate specificities; i.e., the former is an SE synthase, and the latter is a TG synthase. To clarify the intracellular localization of these two enzymes, each EGFP-fused protein was expressed in *A. limacinum* mh0186. First, EGFP fused to the N-terminal of DGAT2C (GFP-DGAT2C) and that to the C-terminal of DGAT2C (DGAT2C-GFP) were expressed in *dgat2c* KO (*dgat2c* KO/*gfp-dgat2c* OE and *dgat2c* KO/*dgat2c-gfp* OE), and each SE productivity was examined to verify the effect of EGFP fusion on enzyme activity. GFP-DGAT2C showed a similar SE productivity to the intact DGAT2C; however, DGAT2C-GFP decreased the SE productivity, indicating that the fusion of EGFP to the C-terminal end may affect the function and stability of DGAT2C (Fig. S6). Therefore, the localization of DGAT2 was examined using N-terminal EGFP-fusion proteins in this study.

**Fig 4:**
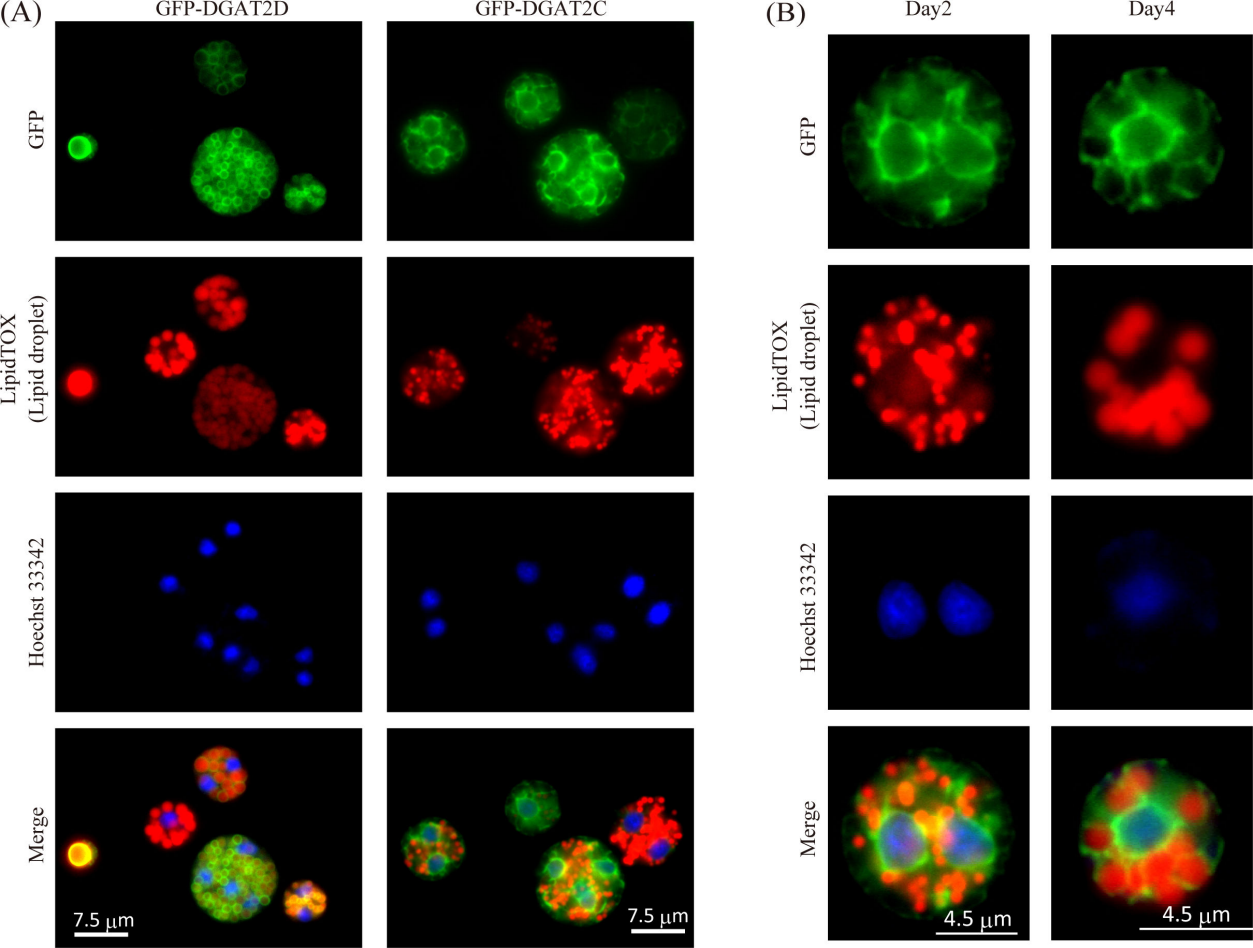
Intracellular localization of GFP-DGAT2D and GFP-DGAT2C in *A. limacinum* mh0186. (**A**) Localization of GFP-DGAT2D and GFP-DGAT2C. EGFP was fused with the N-terminal of DGAT2D or DGAT2C, respectively. GFP-DGAT2D- and GFP-DGAT2C-expressing constructs were integrated into the genomic DNA of *A. limacinum* mh0186 and stably expressed by the ubiquitin promoter. (**B**) Localization of DGAT2C in different culture periods. LDs were stained with high-content screening LipidTOX Red neutral lipid stain, and Hoechst 33342 was used for staining nuclei.

It was found that N-terminal EGFP-DGAT2D localized to the LD, while N-terminal EGFP-DGAT2C localized to the ER, which is the organelle encircling the nuclei (Fig. 4A). Although LDs grew and became larger on day 4, localization of N-terminal EGFP-DGAT2C on the ER was unchanged on days 2 and 4 of culture (Fig. 4B). The localization of N-terminal EGFP-DGAT2C is very similar to that of EGFP with ER retention signal (23), and DGAT2C was estimated to localize to the ER by MulocDEEP localization prediction based on its primary structure (30). These results indicate that TG and SE are synthesized at different organelles by DGAT2D and DGAT2C, respectively, in *A. limacinum* mh0186.

### Analysis of N-terminal deletion mutants of DGAT2C

**Fig 5:**
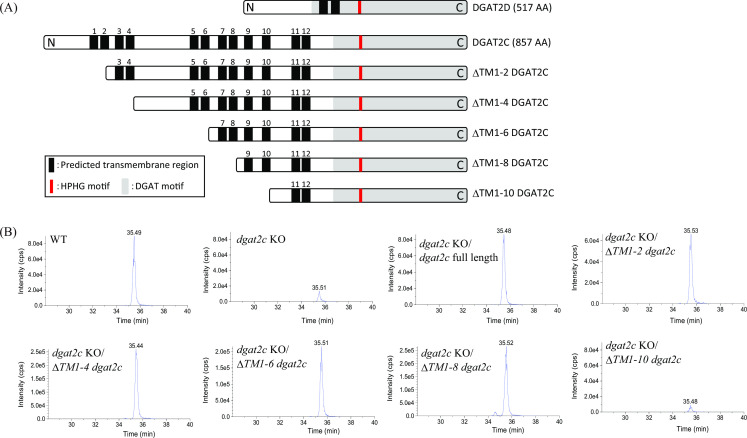
SE synthase activity in N-terminal deletion mutants of DGAT2C. (**A**) Structural schematics of DGAT2D and DGAT2C showing the putative transmembrane regions, catalytic HPHG motif, and DGAT motif. In comparison with DGAT2D, DGAT2C has unique 12 putative transmembrane regions. To evaluate the function of the N-terminal of DGAT2C, each of the two putative transmembrane regions was deleted from the N-terminal in a graded manner to make a series of deletion mutants, ΔTM1-2, ΔTM1-4, ΔTM1-6, ΔTM1-8, and ΔTM1-10 DGAT2C. (**B**) Chromatogram of C22:6-CE of each strain detected by LC-ESI MS/MS analysis. A series of N-terminal-truncated mutants of *dgat2c* were reintroduced into the *dgat2c* KO strain, and their SE synthetic abilities were verified using LC-ESI MS/MS (MRM mode).

**Fig 6:**
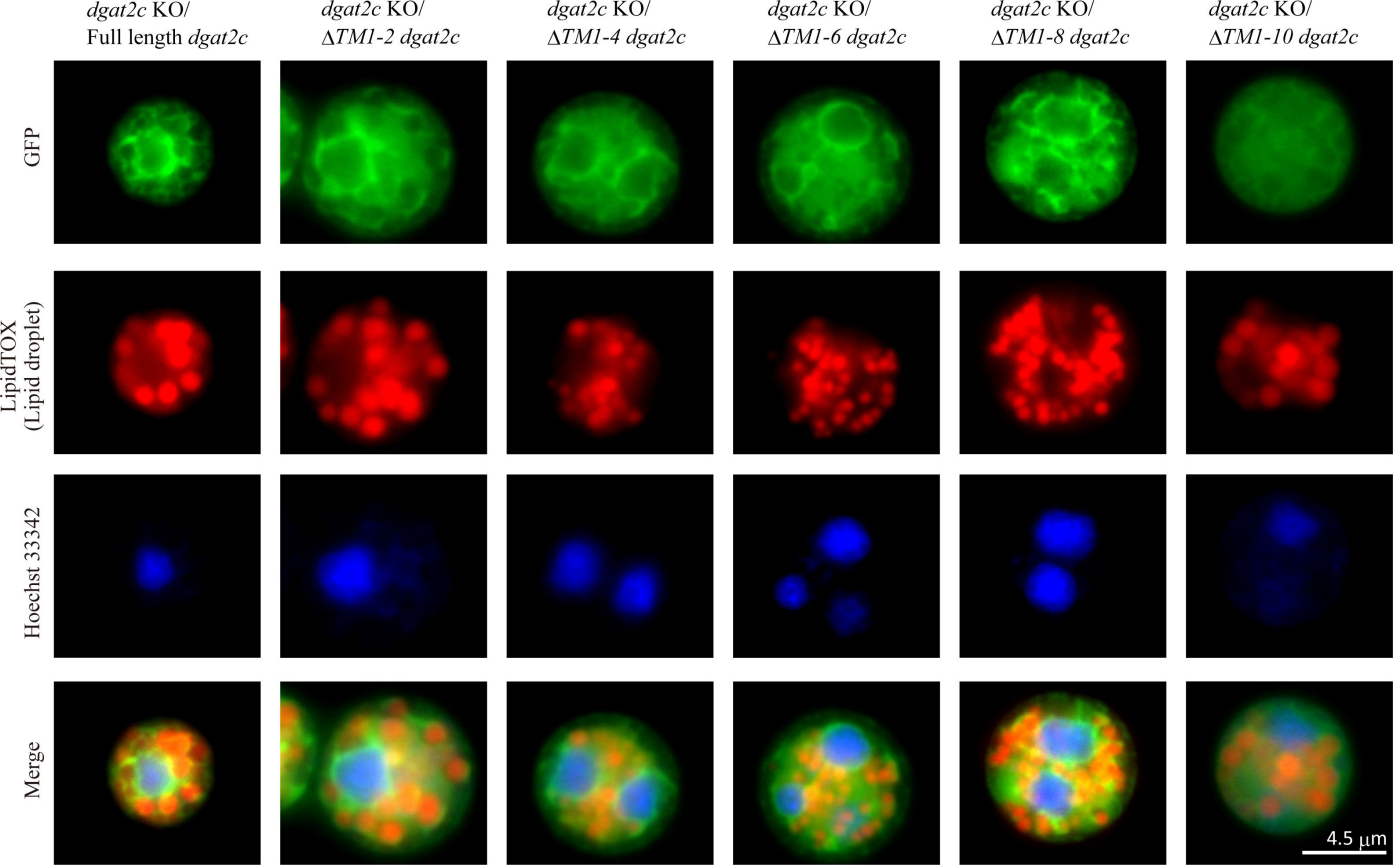
Localization of N-terminal deletion mutants of DGAT2C in *A. limacinum* mh0186. N-terminal EGFP-fused, full-length ΔTM1-2, ΔTM1-4, ΔTM1-6, ΔTM1-8, and ΔTM1-10 *dgat2c* were expressed in the *dgat2c* KO strain of *A. limacinum* mh0186. LDs were stained with high-content screening LipidTOX red neutral lipid stain, and Hoechst 33342 was used for staining nuclei.

To disclose the molecular mechanism which makes the difference in substrate specificity and localization of DGAT2C and DGAT2D, we generated five N-terminal deletion mutants in which two putative transmembrane regions were deleted from the N-terminal of DGAT2C (Fig. 5) because DGAT2C possesses 10 more putative transmembrane regions on the N-terminal side than DGAT2D (Fig. 5A; Fig. S7A and B). We expressed these deletion mutants of DGAT2C in *dgat2c* KO of *A. limacinum* mh0186 (ΔTM1-2, ΔTM1-4, ΔTM1-6, ΔTM1-8, and ΔTM1-10 *dgat2c* strains). LC-ESI MS/MS analysis revealed that production of C22:6-CE (Fig. 5B) and C22:6-Δ7-SE (Fig. S8) was maintained in deletion mutant strains except for the ΔTM1-10 *dgat2c* strain, indicating that the sequence containing the 9th and 10th putative transmembrane regions is indispensable for the SE synthase activity of DGAT2C. ΔTM1-2, ΔTM1-4, ΔTM1-6, and ΔTM1-8 DGAT2C localized to the ER, such as full-length DGAT2C (Fig. 6). Alternatively, ΔTM1-10 DGAT2C did not localize to the ER (Fig. 6) and showed similar localization of EGFP alone, which localizes at cytosol (23). The sequence containing the 9th and 10th putative transmembrane regions is essential for SE production and ER localization of DGAT2C in *A. limacinum* mh0186.

### The function of the N-terminal region of DGAT2C

**Fig 7:**
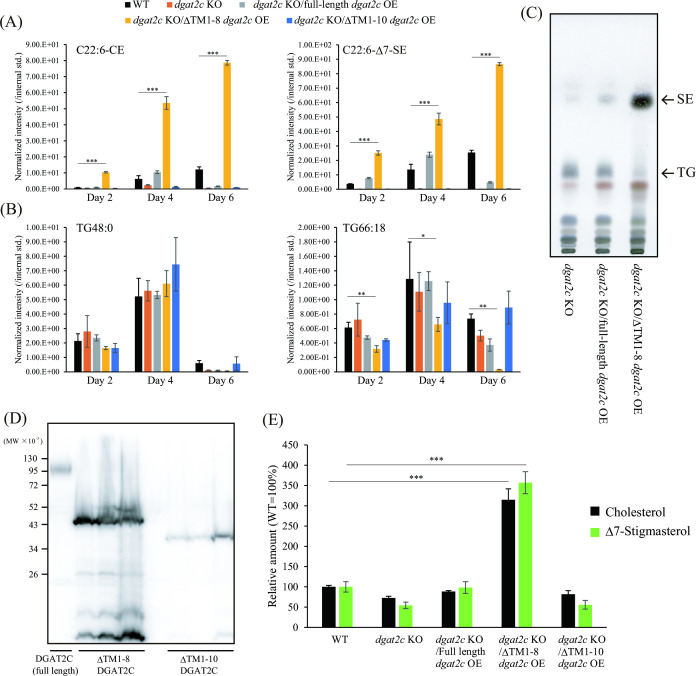
Increase in SE and total sterol amounts in *A. limacinum* mh0186 by ΔTM1-8 DGAT2C expression. (**A**) Quantification of C22:6-CE (left panel) and C22:6-Δ7-SE (right panel) of WT, *dgat2c* KO, *dgat2c* KO/full-length *dgat2c* OE, *dgat2c* KO/ΔTM1-8 *dgat2c* OE, and *dgat2c* KO/ΔTM1-10 *dgat2c* OE strains. (**B**) Quantification of TG48:0 (C16:0/C16:0/C16:0, left panel) and TG66:18 (C22:6/C22:6/C22:6, right panel) of the same strains described in panel **A**. The cellular C22:6-CE, C22:6-Δ7-SE, TG48:0, and TG66:18 levels of each strain were measured using LC-ESI MS/MS (MRM mode) at the indicated time points. The full-length, ΔTM1-8, and ΔTM1-10 *dgat2c* were reintroduced into the *dgat2c* KO strain. The peak intensity of each lipid was normalized with that of the internal standard. (**C**) TLC analysis showing the neutral lipid profiles of *dgat2c* KO, *dgat2c* KO/full-length *dgat2c* OE, and *dgat2c* KO/ΔTM1-8 *dgat2c* OE strains. Lipid extracts were applied to a TLC plate that was developed with hexane/diethyl ether/acetic acid = 80/20/1 (vol/vol/vol). Neutral lipids were visualized by spraying copper sulfate solution. (**D**) Western blotting showing the protein expression levels of full-length DGAT2C, ΔTM1-8 DGAT2C and ΔTM1-10 DGAT2C. The molecular mass of full-length, ΔTM1-8 and ΔTM1-10 DGAT2C is estimated to be 98.8, 53.1, and 39.1 kDa, respectively. Three independent ΔTM1-8- and ΔTM1-10 *dgat2c*-expressing strains were obtained and applied to Western blotting analysis. (**E**) Quantification of total sterol of the WT, *dgat2c* KO, *dgat2c* KO/full-length *dgat2c* OE, *dgat2c* KO/ΔTM1-8 *dgat2c* OE, and *dgat2c* KO/ΔTM1-10 *dgat2c* OE strains. The total of the free form of sterols was measured using LC-APCI MS/MS (MRM mode) after releasing sterols from SE by methanolysis. The relative amount with the WT as 100% of cholesterol and Δ7-stigmasterol is calculated. Data represent means ± SDs of three separate experiments. Statistical significance is indicated as follows: *P < 0.05, **P < 0.01, and ***P < 0.001.

This study indicates that the N-terminal region containing the first to the eighth putative transmembrane regions was not required for SE production and ER localization of DGAT2C in *A. limacinum* mh0186. To elucidate the function of the N-terminal region, C-terminal *flag*-tagged, full-length ΔTM1-8 and ΔTM1-10 *dgat2c* were expressed in the *dgat2c* KO of *A. limacinum* mh0186 (full-length *dgat2c*, ΔTM1-8 *dgat2c*, and ΔTM1-10 *dgat2c* strains, respectively), and their SE production and protein expression levels were examined. Interestingly, the amounts of SE markedly increased in the ΔTM1-8 *dgat2c* strain compared to those in the WT and full-length *dgat2c* strain during the culture period (Fig. 7A). However, the ΔTM1-10 *dgat2c* strain showed no production of C22:6-CE and C22:6-ΔSE (Fig. 7A). Conversely, the amount of TG comprising three DHA (TG66:18) decreased significantly in the ΔTM1-8 *dgat2c* strain (Fig. 7B), suggesting that DHA is used for SE synthesis instead of TG because of high SE synthase activity in the ΔTM1-8 *dgat2c* strain. The increase in SE and the decrease in TG in the ΔTM1-8 *dgat2c* strain were also confirmed using TLC analysis (Fig. 7C). No significant difference was observed in the EE level when the ΔTM1-8*,* full-length, or ΔTM1-6 *dgat2c* was expressed in *S. cerevisiae* (Fig. S9A). Western blot analysis revealed that the ΔTM1-8 DGAT2C expression was significantly higher than that of full-length or ΔTM1-10 DGAT2C when expressed in the *dgat2c* KO strain of *A. limacinum* (Fig. 7D). These results suggested that the increase in SE production in the ΔTM1-8 *dgat2c* strain is at least in part due to high DGAT2C expression in *A. limacinum*. An increase in DGAT2C expression and SE production was not observed in the ΔTM1-6 DGAT2C strain, and thus, the amino acid sequence containing the seventh and eighth transmembrane regions may be important for controlling the expression of DGAT2C in *A. limacinum* (Fig. S9B and C).

Next, the amount of free sterol level in the ΔTM1-8 *dgat2c* strain was measured by LC-APCI MS/MS analysis before and after methanolysis with hydrochloric acid (Fig. S10A). In contrast to SE, the amount of free sterol without methanolysis decreased in the ΔTM1-8 *dgat2c* strain compared to that in the WT or other mutant strains (Fig. S10B), suggesting that free sterol pools were used more for SE synthesis in the ΔTM1-8 *dgat2* strain. Alternatively, the cholesterol and Δ7-stigmasterol amounts in this mutant strain drastically increased after methanolysis, reaching three times higher levels than those of the WT strain (Fig. 7E). This result indicates that the total sterol amount drastically increased in the ΔTM1-8 *dgat2* strain compared to that in the WT strain.

**Fig 8:**
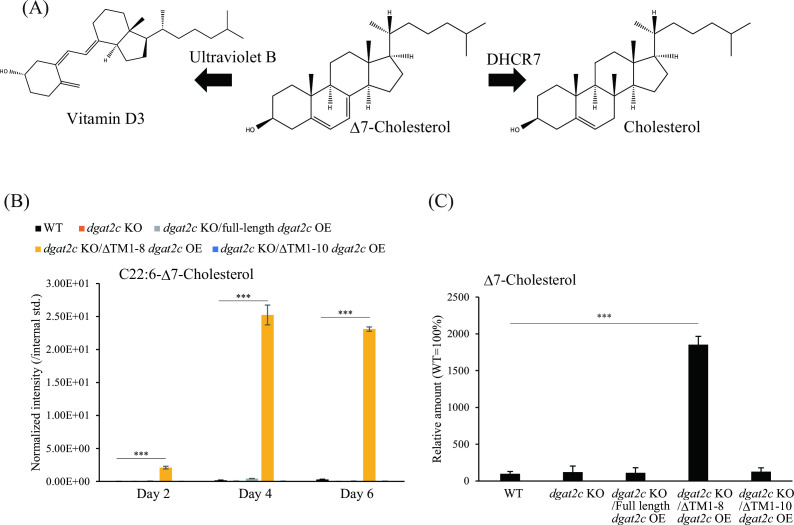
Production of provitamin D3 ester (Δ7-cholesterol ester) in *A. limacinum* mh0186 by ΔTM1-8 DGAT2C expression. (**A**) Correlation between cholesterol, Δ7-cholesterol, and vitamin D3 in the synthetic pathway of cholesterol. The double bond at the Δ7 position in ring B of Δ7-cholesterol is reduced by DHCR7 to generate cholesterol. Vitamin D3 is generated from Δ7-cholesterol by irradiation of ultraviolet B. (**B**) Quantification of Δ7-cholesterol ester composed of C22:6 (C22:6-Δ7-cholesterol) of the WT, *dgat2c* KO, *dgat2c* KO/full-length *dgat2c* OE, *dgat2c* KO/ΔTM1-8 *dgat2c* OE, and *dgat2c* KO/ΔTM1-10 *dgat2c* OE strains. The cellular C22:6-Δ7-cholesterol level of each strain was measured using LC-ESI MS/MS (MRM mode) at the indicated time points. The peak intensity of each lipid was normalized with that of the internal standard. (**C**) Quantification of total Δ7-cholesterol. The total amount of Δ7-cholesterol was measured using LC-APCI MS/MS (MRM mode) after releasing sterol from SE by methanolysis. The relative amount with the WT as 100% is calculated. Data represent means ± SDs of three separate experiments. Statistical significance is indicated as follows: ***P < 0.001.

In the cholesterol synthesis pathway, Δ7-cholesterol (7-dehydrocholesterol) is a precursor of cholesterol (Fig. 8A). The C7-C8 double bond in the B ring of Δ7-cholesterol is reduced by 7-dehydrocholesterol reductase (DHCR7) to generate cholesterol. Because Δ7-cholesterol is converted to vitamin D3 by ultraviolet B, Δ7-cholesterol is called provitamin D3. Δ7-Cholesterol and Δ7-cholesterol-ester were rarely detected in the WT of *A. limacinum* during the course of the culture period (Fig. 8B and C; Fig. S11A). We found that a substantial amount of Δ7-cholesterol-ester containing C22:6 (C22:6-Δ7-cholesterol) was produced in the ΔTM1-8 *dgat2c* strain cultured for 4 and 7 days (Fig. 8B). The *m*/*z* 712.5, which is derived from C22:6-Δ7-cholesterol, was detected only in the ΔTM1-8 *dgat2c* strain (Fig. S11A), and the structure of C22:6-Δ7-cholesterol was confirmed by the MS/MS analysis, in which fragment ions 145 and 159 derived from double bonds at Δ5 and Δ7 of the B-ring in Δ7-cholesterol were detected (26) (Fig. S11B), respectively. Consistent with the accumulation of C22:6-Δ7-cholesterol, the total amount of Δ7-cholesterol drastically increased in the ΔTM1-8 *dgat2c* strain (Fig. 8C). These results indicated that ΔTM1-8 DGAT2C expression is useful for high production of Δ7-stigmasterol, cholesterol and Δ7-cholesterol that are rarely synthesized in *A. limacinum*.

## DISCUSSION

This study indicated that two DGAT2 homologs in *A. limacinum* mh0186 use different acceptor substrates; i.e., DGAT2C and DGAT2D use sterols and DG as acceptor substrates to generate SE and TG, respectively. Rau et al. reported that AlASATb of *A. limacinum* SR21, whose sequence is very close to that of DGAT2C of *A. limacinum* mh0186, could be an SE synthase by knockout and overexpression of *alasatb* in *A. limacinum* SR21 (27). In addition to the genetic approach in *A. limacinum*, we showed that DGAT2C synthesizes SE from acyl-CoA and sterols by heterologous expression of *dgat2c* in budding yeast using an *in vitro* enzyme assay. Furthermore, we successfully developed a method to increase sterol production in the thraustochytrid by elucidating the function of the DGAT2C-specific N-terminal region. The mutant strain expressing ΔTM1-8 DGAT2C enabled the production of cholesterol and Δ7-stigmasterol more than three times that of WT *A. limacinum* (Fig. 7E). These results are expected to be a clue of the practical application of sterol production using thraustochytrids in the future.

The growth characteristic of the *dgat2d* KO was different from that of the *dgat2a* and *dgat2c* KO mutant strains (Fig. S3). WT cells showed a maximum TG level when they reached the stationary phase (day 4), followed by a decrease in the decline phase (day 6) (Fig. 7B; Fig. S3A and B), suggesting that TG is stored in LD as an alternative energy resource to survive after external carbon sources such as glucose are exhausted in the medium (Fig. S3C and D). Thus, we expected that the loss of TG in *dgat2d* KO may cause a difference in OD600 after the stationary phase. However, the OD600 values of *dgat2d* KO had already decreased in the early stages of the culture, despite the presence of sufficient glucose in the medium (Fig. 2A and B). Our result suggests that TG may have other important biological functions besides simply being a nutrient storage for *A. limacinum*, but the details are unknown.

Fluorescent microscopy analysis using EGFP-fused DGAT2 homologs revealed that DGAT2C is entirely different in localization from DGAT2D, i.e., the former localizes to the ER and the latter to the LDs in *A. limacinum* mh0186 (Fig. 4). LD proteins are divided into Classes I and II (31, 32). Class I proteins originally localize to the ER and then move to the LDs during the formation and maturation of LDs. Alternatively, Class II proteins directly localize to the LDs from the cytoplasm. DGAT2D is likely a Class I protein because it is predicted to localize to the ER from its primary structure. Although DGAT2D is speculated to have two transmembrane regions, as shown in Fig. 5A, DGAT2D appears to be a monotopic protein that enters the membrane region but does not penetrate to the opposite side (Fig. S12A). Monotopic proteins are stably incorporated into the membrane via hydrophobic hairpin domain, forming a topology in which the soluble domain is exposed toward one side of the membrane. In many cases, proline residues at the midpoint of the hydrophobic regions break the helix structure to form a hairpin domain. The presence of proline at the center of the helical domain of DGAT2D supports that DGAT2D has a hydrophobic hairpin domain (Fig. S12A) and localizes to the LDs as a monotopic protein. In contrast to DGAT2D, DGAT2C localized to the ER but not to the LDs during the culture period (Fig. 4A and B). Although ΔTM1-8 DGAT2C localized to the ER, ΔTM1-10 DGAT2C did not (Fig. 6), suggesting that the 9th and 10th putative transmembrane regions are real transmembrane regions, which enter the ER membrane (Fig. S12B).

In agreement with the study by Rau et al. (27), DHA is the main fatty acid linked to sterols in *A. limacinum* (Fig. S4), suggesting that DGAT2C may prefer DHA-CoA over saturated fatty acyl-CoA. However, the experiment of heterologous expression in yeast revealed that 16:0-EE and 18:0-EE were synthesized by DGAT2C, indicating that DGAT2C has a broad substrate specificity and can utilize not only DHA-CoA but also saturated fatty acyl-CoA. Thus, we wonder why DHA-containing SE was selectively synthesized in *A. limacinum*. Similar to DGAT2C, PC synthase, PLAT1, which is estimated to localize at ER, synthesizes significantly more DHA-containing PCs (PC38:6 and PC44:12) than PCs containing only saturated fatty acids (PC32:0) in *A. limacinum*. However, it has no specificity for fatty acyl-CoA species (33). These results indicate that DHA-CoA is more accessible than palmitoyl-CoA for ER-localized enzymes, leading to preferentially synthesized DHA-containing SE or PC.

The C-terminal region of DGAT2C has a DGAT motif that shows high similarities with DGAT2D (Fig. 5; Fig. S12). The HPHG sequence, which is essential for the activity of DGAT2-like enzymes, is also conserved in the C-terminal DGAT motif in DGAT2C. The third residue (histidine) in the HPGH motif is demonstrated to be integral to the SE synthase activity of DGAT2C (Fig. 3G). This result suggests that the mechanism for synthesizing SE by DGAT2C is partly similar to that of TG by conventional DGAT2, although DGAT2C utilizes sterol instead of DG as an acceptor substrate. As shown in the three-dimensional structure of mammalian ACAT1 that transfers fatty acid from acyl-CoA to cholesterol, transmembrane regions are involved in sterol recognition (34). Thus, we hypothesized that the unique substrate specificity of DGAT2C could be dependent on 12 putative transmembrane regions in the N-terminal region that are not present in DGAT2D (Fig. 5A). In this study, we showed that the 1st to 8th transmembrane regions of DGAT2C are not essential for SE production in *A. limacinum*, suggesting that the 9th to 12th transmembrane regions would be involved in recognizing sterol (Fig. S12B). Importantly, these transmembrane regions are also conserved in DGAT2C of thraustochytrids other than *A. limacinum* (Fig. S13) but not in DGAT2 homologs of other eukaryotes. Thus, DGAT2C is considered a unique SE synthase specifically acquired in thraustochytrids during their evolution.

Surprisingly, the ΔTM1-8 *dgat2c* strain showed a significant increase in SE, leading to a threefold increase in total cholesterol and Δ7-stigmasterol compared to the WT strain of *A. limacinum* mh0186 (Fig. 7E). Furthermore, Δ7-cholesterol and Δ7-cholesterol-SE, which are rarely detected in the WT, were generated in the ΔTM1-8 *dgat2c* strain (Fig. 8B and C). The demand for sterols and SE has been increasing because of their usefulness; thus, a sustainable source for sterol production is required. As described in the Introduction, phytosterols have biological benefits such as anti-inflammatory, anti-oxidant, and anti-cancer activities. Cholesterol and Δ7-cholesterol are a precursor for the steroid hormone ecdysterone, which regulates molting, growth, and immune response in crustaceans (35). In addition, cholesterol can stimulate the synthesis of sex hormones, promote gonadal development, and facilitate crustacean reproduction and fecundity (36). Since the *de novo* synthetic pathway of cholesterol is lost in crustaceans, cholesterol in feed is necessary for shrimp aquaculture (37). Thuraustochytrids are evaluated as a sustainable aquaculture feed alternative to fishmeal and fish oil because they can synthesize n-3PUFA, which is indispensable for the growth of marine fish (38). In addition to fish aquaculture, cholesterol and Δ7-cholesterol production-potentiated thraustochytrid are expected to be used for shrimp aquaculture.

The major reason for the increase in SE content when ΔTM1-8 DGAT2C was expressed in *dgat2c*-deficient *A. limacinum* was likely due to the increase in ΔTM1-8 DGAT2C expression at the protein level (Fig. 7D). This could be due to the presence of the region that negatively regulates the expression or promotes the degradation of DGAT2C in the first to the eighth putative transmembrane regions. We might be able to rule out the possibility that the enzymatic activity of DGAT2C *per se* was increased by deleting the putative transmembrane region since no significant increase in SE synthesis was observed in ΔTM1-8 *dgat2c* expression in budding yeast (Fig. S9A).

Δ7-Cholesterol, provitamin D3, is converted to vitamin D3 by ultraviolet B in sunlight (39). Fractures and bone loss have been linked to vitamin D deficiency. Severe vitamin D deficiency significantly increases the risk of infection and many other diseases (40). Approximately 40% of Europeans are suggested to have a vitamin D deficiency, and 13% have a severe deficiency (41). Vitamin D has rare side effects, a wide safety margin, and pathophysiological links with energy homeostasis, immune system function, and endocrine regulation (40). The individual patient data meta-analysis of randomized placebo-controlled trials to evaluate vitamin D’s effect on cancer mortality indicated that daily vitamin D supplementation could reduce cancer mortality by 12% (42). Thus, vitamin D is an important, safe, and inexpensive adjunctive therapy for multiple diseases, particularly cancer. Supplementation with vitamin D is recommended because sunlight exposure and dietary intake are often insufficient to maintain optimal vitamin D status (40). The growing awareness of vitamin D has led to an increase in the use of vitamin D supplements and the expansion of its market (43). Recent attempts have been made to produce vitamin D3 using thraustochytrid because of its high sterol productivity. For the production of Δ7-cholesterol, DHCR7, which converts Δ7-cholesterol to cholesterol (Fig. 8A), is deleted in *Aurantiochytrim* sp (44). In this study, we successfully developed a new method that significantly increases the production of Δ7-cholesterol. It is expected to lead to a higher production of vitamin D3 in *A. limacinum* by combining these different strategies synergistically.

Prediction of localization of DHCR7 based on its sequence in *A. limacinum* indicates that it is likely to localize to the ER similarly with DGAT2C. Δ7-Cholesterol would be converted to Δ7-cholesterol ester by ΔTM1-8 DGAT2C before being converted to cholesterol by DHCR7 because of the high expression of ΔTM1-8 DGAT2C, then Δ7-cholesterol ester finally accumulates in LDs where no reduction in Δ7-cholesterol occurs. ΔTM1-8 DGAT2C expression in *A. limacinum* produced Δ7-cholesterol ester and unknown SE that was eluted before C22:6-Δ7-SE on LC (Fig. S8), indicating that ΔTM1-8 DGAT2C could utilize intermediate(s) other than Δ7-cholesterol to generate their SE.

This study disclosed more detailed properties and the localization of DGAT2C, a unique SE synthase in thraustochytrids. We found that the expression of a mutant enzyme that appropriately truncated the N-terminal region of DGAT2C could increase the SE content and, thus, the total sterol content in *A. limacinum* mh0186. This finding is expected to be one of the cornerstones for future industrial production of sterols using thraustochytrids.

## SUPPLEMENTAL MATERIAL

Supplemental tables and figures aem.01001-23-s0001.pdf This file includes supplemental tabels (S1 and S2) and supplemental figures (S1 - S13).

ASM does not own the copyrights to Supplemental Material that may be linked to, or accessed through, an article. The authors have granted ASM a non-exclusive, world-wide license to publish the Supplemental Material files. Please contact the corresponding author directly for reuse.
