# Supplementary material for: Characterization of thraustochytrid-specific sterol O-acyltransferase: modification of DGAT2-like enzyme to increase the sterol production in Aurantiochytrium limacinum mh0186
Source: Appl Environ Microbiol. 2023 Oct 24;89(11):e01001-23. doi: 10.1128/aem.01001-23 (PMC10686087; doi:10.1128/aem.01001-23)
Supplement: Supplemental tables and figures — This file includes supplemental tabels (S1 and S2) and supplemental figures (S1 - S13). [file aem.01001-23-s0001.pdf]

# Table S1. Oligonucleotide primers used in this study.

| Primer name | Sequence | Purpose |
|---|---|---|
| DGAT2A5UTR-S | GAATCAGGTCCGAATCCTGTGGCTCG | |
| DGAT2A3UTR-A | GTGTGCAGACGTCGGAGAGGAGAATGGAC | |
| DGAT2A3UTR-HygR-S | GATCTCACATTAATTGCGTCAAAGCGTTGCTAC | |
| DGAT2A5UTR-HygR-A | GGCAGAAGAGACACACAATACGTTGGACTCTAG | *dgat2a* KO |
| DGAT2AHygR-S | CTAGAGTCCAACGTATTGTGTGTCTCTTCTTGCC | |
| DGAT2AHygR-A | CAGTAGCAACGCTTTGACGCAATTAATGTGAGAT | |
| DGAT2D5UTR-S | TTCTTTCTGTTTACGTGTTTCCACATTTCTCG | |
| DGAT2D3UTR-A | GTACTGCAAAGAAGAAGCCAAGCTCCATCGTA | |
| DGAT2D3UTR-NeoR-S | TAGATCTCACATTAATTGCGTTAGCAACACTACTACAAAATTTG[illegible] | |
| DGAT2D5UTR-NeoR-A | CTGCGGCAGATCCAGATCTGAGCAAGCATTGAGTACCAGGA | |
| NeoR-S | CCTGGTACTCAATGCTTGCTCACATCTGGATCTGCCGCAGC | *dgat2d* KO |
| NeoR-A | TCAAATTTTGTAGTAGTGTTGCTAACGCAATTAATGTGAGATCT | |
| DGAT2DORF-S | AGAATTCATGCCTGTTCTTATTGCACTTC | |
| DGAT2DORF-A | GCTCGAGTTACTTCTGAATTAAAAGAGAAC | |
| DGAT2C5UTR-S | GGACTTCCTACTTTTGTATTAATGGCACTAGCA | |
| DGAT2C3UTR-A | TCTTCGCCTTCTTACCCATCGTGTGTTACTC | |
| DGAT2C3UTR-HygR-S | ACATTAATTGCGTGTGCGCTCTAATCAACAA | |
| DGAT2C5UTR-HygR-A | AGATCCAGATCTGACCTCGACGTCGCT | *dgat2c* KO |
| HygR-S | AAGCGACGTCGAGGTCAGATCTGGAT | |
| HygR-A | GTTGTTGATTAGAGCGCACACGCAATTAATG | |
| A.L_Vector_Rv | TTTTGTTGGCTAGTGTTGC | |
| A.L_Vector_Fw | GATTACAAGGACGACGATGACAAGtagAACTAAGCTATCTGTAG | *dgat2c-flag* OE |
| DGAT2C FLAG-S | CACTAGCCAACAAAAATGGCGGAGGGCGTCGAGGG | |
| DGAT2C FLAG-A | GTCGTCCTTGTAATCATAGTCGATGAAGTCGAGCTC | |
| pYESCTCF-S | GATTACAAGGACGACGATG | |
| pYESCTCF-A | GGATCCGAGCTCGGTACC | |
| pYES-DGAT2D-S | ACCGAGCTCGGATCCATGTCTTCAAGCCCCGCC | pYES-*dgat2d* |
| pYES-DGAT2D-A | GTCGTCCTTGTAATCCTTCTGAATTAAAAGAGAACC | pYES-*dgat2c* |
| pYES-DGAT2C-S | ACCGAGCTCGGATCCATGGCGGAGGGCGTCGAGGG | |
| pYES-DGAT2C-A | GTCGTCCTTGTAATCATAGTCGATGAAGTCGAGCTC | |
| DGAT2C-H644A-S | TCCACCCTgcTGGTATCTTC | pYES-*dgat2c* H644A |
| DGAT2C-H644A-A | GAAGATACCAgcAGGGTGGA | |
| DGAT2C-H642A-S | TTGCTTTTCgcCCCTCATGG | pYES-*dgat2c* H642A |
| DGAT2C-H642A-A | CCATGAGGGgcGAAAAGCAA | |
| pENUG-nDGAT2C-F | GCAACACTAGCCAACATGGCGGAGGGCGTCGA | |
| pENUG-nDGAT2C-R | gcccttgctcaccatATAGTCGATGAAGTCGAGCT | |
| C-GFP-S | atggtgagcaagggcgaggagct | *dgat2c-egfp* |
| C-GFP-A | GTTGGCTAGTGTTGCTTAGATCGCTTGC | |
| pENUG-cDGAT2C-F | gacgagctgtacaagATGGCGGAGGGCGTCGA | |
| pENUG-cDGAT2C-R | ACAGATAGCTTAGTTTTAATAGTCGATGAAGTCGA | |
| N-GFP-S | AACTAAGCTATCTGTAGTATGTGCTATTCCCG | *egfp-dgat2c* |
| N-GFP-A | cttgtacagctcgtccatgccgag | |
| GFP-DGAT2C-TMd1 | gacgagctgtacaagATTGGACATCTAGTCCAC | ΔTM1-2 egfp-dgat2c |
| GFP-DGAT2C-TMd2 | gacgagctgtacaagAGCAACAGCTCTCAATCTTC | ΔTM1-4 egfp-dgat2c |
| GFP-DGAT2C-TMd3 | gacgagctgtacaagCGTTTGCGGCATTTTCATC | ΔTM1-6 egfp-dgat2c |
| GFP-DGAT2C-TMd4 | gacgagctgtacaagCGATTCGACCAAAAGGAG | ΔTM1-8 egfp-dgat2c |
| GFP-DGAT2C-TMd5 | gacgagctgtacaagGAGGGATGGCGGCAGAG | ΔTM1-10 egfp-dgat2c |
| pAlim-TMd3-2C-infu-S | CACTAGCCAACAAAAATGCGTTTGCGGCATTTTCATC | ΔTM1-6 dgat2c flag |
| pAlim-TMd4-2C-infu-S | CACTAGCCAACAAAAATGCGATTCGACCAAAAGGAG | ΔTM1-8 dgat2c flag |
| pAlim-TMd5-2C-infu-S | CACTAGCCAACAAAAATGGAGGGATGGCGGCAGAG | ΔTM1-10 dgat2c flag |
| realtime2AF | ATGACGACGAGGTTGTTGCTCTG | |
| realtime2AR | GAGAAAGCCACTCCATGACTGGA | |
| realtime2CF | ACTCATGCTGTGCTACCCCTCAG | Quantitative real time PCR |
| realtime2CR | TCCATTGAATGACATCACGCATC | |
| realtime2DF | TGGGCACGAAATGTTCAGGAG | |
| realtime2DR | AGAGCACGGCCCTTTAATTCATC | |

Table S2. MRM conditions for the lipid analysis by using LC-ESI MS/MS.

| | Compound | Q1 | Q3 |
|---|---|---|---|
| Free Sterol | Cholesterol 1 | 369.4 | 161.3 |
| | Cholesterol 2 | 369.4 | 147.4 |
| | Ergosterol 1 | 379.3 | 159.2 |
| | Ergosterol 2 | 379.3 | 145.2 |
| | Δ7-Stigmasterol 1 | 393.4 | 159.4 |
| | Δ7-Stigmasterol 2 | 393.4 | 145.4 |
| | Δ7-cholesterol 1 | 367.4 | 159.3 |
| | Δ7-cholesterol 2 | 367.4 | 145.4 |
| Sterol Ester | C16:0-EE | 652.3 | 379.3 |
| | C16:1-EE | 652.3 | 379.3 |
| | C18:0-EE | 652.3 | 379.3 |
| | C18:1-EE | 652.3 | 379.3 |
| | C22:5-EE | 726.3 | 379.3 |
| | C22:6-EE | 724.3 | 379.3 |
| | C16:0-CE | 642.6 | 369.4 |
| | C22:5-CE | 716.6 | 369.4 |
| | C22:6-CE | 714.6 | 369.4 |
| | C16:0-Δ7-SS | 666.4 | 393.4 |
| | C22:5-Δ7-SS | 740.4 | 393.4 |
| | C22:6-Δ7-SS | 738.4 | 393.4 |
| | C16:0-Δ7-CE | 640.6 | 367.4 |
| | C22:5-Δ7-CE | 714.6 | 367.4 |
| | C22:6-Δ7-CE | 712.6 | 367.4 |
| | d7-C16:0-CE (IS) | 649.6 | 376.4 |
| PC | PC32:0 | 734.5 | 184.1 |
| | PC38:6 | 806.5 | 184.1 |
| | PC44:12 | 878.5 | 184.1 |
| | PC22:0 (IS) | 594.4 | 184.1 |
| Diacylglycerol | DG32:0 16:0/16:0 | 586.5 | 313.5 |
| | DG38:6 22:6/16:0 | 658.5 | 313.5 |
| | DG44:12 22:6/22:6 | 730.5 | 385.5 |
| | DG24:0 12:0/12:0 (IS) | 474.4 | 257.2 |

| | Compound | Q1 | Q3 |
|---|---|---|---|
| Triacylglycerol | TG48:0 (16:0/16:0/16:0) | 824.5 | 551.5 |
| | TG54:6 (16:0/22:6/16:0) | 896.5 | 623.5 |
| | TG60:12 (22:6/22:6/16:0) | 968.5 | 623.5 |
| | TG66:18 (22:6/22:6/22:6) | 1040.5 | 695.5 |
| | TG42:0(14:0/14:0/14:0) | 740.5 | 495.5 |
| | TG44:0(16:0/14:0/14:0) | 768.5 | 495.5 |
| | TG48:1 (16:1/16:0/16:0) | 822.5 | 549.5 |
| | TG48:2 (16:1/16:1/16:0) | 820.5 | 547.5 |
| | TG48:3 (16:1/16:1/16:1) | 818.5 | 545.5 |
| | TG46:1(18:1/14:0/14:0) | 794.5 | 495.5 |
| | TG46:0(18:0/14:0/14:0) | 796.5 | 495.5 |
| | TG46:0(16:0/16:0/14:0) | 796.5 | 523.5 |
| | TG48:1(18:1/16:0/14:0) | 822.5 | 523.5 |
| | TG48:0(18:0/14:0/16:0) | 824.5 | 523.5 |
| | TG50:2(18:1/18:1/14:0) | 848.5 | 549.5 |
| | TG50:1(18:0/18:1/14:0) | 850.5 | 549.5 |
| | TG50:1(18:1/16:0/16:0) | 850.5 | 551.5 |
| | TG50:0(18:0/16:0/16:0) | 852.5 | 579.5 |
| | TG52:2(18:1/18:1/16:0) | 876.5 | 577.5 |
| | TG52:1(18:1/18:0/16:0) | 878.5 | 579.5 |
| | TG52:0(18:0/18:0/16:0) | 880.5 | 579.5 |
| | TG54:3(18:1/18:1/18:1) | 902.5 | 603.5 |
| | TG54:2(18:2/18:0/18:0) | 904.5 | 607.5 |
| | TG54:2(18:1/18:1/18:0) | 904.5 | 605.5 |
| | TG54:1(18:1/18:0/18:0) | 906.5 | 607.5 |
| | TG54:0(18:0/18:0/18:0) | 908.5 | 607.5 |
| | TG36:0(12:0/12:0/12:0) (IS) | 656.5 | 439.4 |

## Supplemental figure legends

**Fig. S1. Knockout strategy for *dgat2a, dgat2c,* and *dgat2d* in *A. limacinum* mh0186**

Schematics of DNA constructs for the disruption of (A) *dgat2a,* (B) *dgat2c,* and (C) *dgat2d* in *A. limacinum* mh0186 by homologous recombination. Confirmation of gene disruption of (D) *dgat2a*, (E) *dgat2c*, and (F) *dgat2d* KO by genomic PCR. PCR product size of the WT and gene-disrupted strains are indicated at the top or bottom of (A), (B), and (C), respectively.

**Fig. S2. DG level of *dgat2a*, *dgat2c,* and *dgat2d* KO strains**

(A) Quantification of each molecular species of DG by LC-ESI MS/MS analysis (MRM mode) of WT, *dgat2a*, *dgat2c*, and *dgat2d* KO strains harvested from 3-day cultures. The peak intensity of each lipid was normalized with dry cell weight (DCW). Data represent means ± SD of three separate experiments. (B) TLC analysis showing the neutral lipid profiles of WT and *dgat2d* KO strains. Total lipids were extracted from cells cultured for 3 days. Lipids were applied to a TLC plate that was developed with hexane/diethyl ether/acetic acid = 50/50/1 (v/v/v). Neutral lipids were visualized by spraying copper sulfate solution.

**Fig. S3. Proliferative properties of *dgat2a, dgat2c, and dgat2d* KO strains**

(A) Growth curves for the WT and *dgat2d* KO strain. (B) Glucose consumption rates of WT and *dgat2d* KO strain. (C) Growth curves for the WT, *dgat2a* KO, and *dgat2c* KO strains. (D) Glucose consumption rates of the WT, *dgat2a* KO, and *dgat2c* KO strains. OD600 and glucose concentration of each strain were measured at the indicated time points. Data represent means ± SD of three separate experiments.

**Fig. S4. Quantification of sterol ester containing C16:0 or C22:6 in *A. limacinum* mh0186**

C16:0 (palmitic acid) and C22:6 (DHA) content in Δ7-stigmasterol or cholesterol ester in *A. limacinum*. Lipid extracts of the WT, *dgat2c* KO, and *dgat2c* KO/*dgat2c* OE (revertant) strains that were collected on day five of culture were applied to LC-ESI MS/MS (MRM mode) to measure the amount of SE containing C16:0 or C22:6. The peak intensity of each lipid was normalized with that of the internal standard. Data represent means ± SD of three separate experiments.

**Fig. S5. Quantification of TG of *dgat2d* or *dgat2c* expressing yeast**

*Saccharomyces cerevisiae* was transfected with a pYES vector harboring *dgat2d* or *dgat2c* genes derived from *A. limacinum* mh0186. TG containing C14:0, C16:0, C16:1, C18:0, and C18:1 of the mock, pYES-*dgat2d*-, and pYES-*dgat2c*-transfected yeast were measured using LC-ESI MS/MS (MRM mode). The peak intensity of each lipid was normalized with that of the internal standard. Data represent means ± SD of three separate experiments.

**Fig. S6. Effect of N- or C-terminal GFP fusion on the SE synthetic activity of DGAT2C**

EGFP was fused with N-terminal (e*gfp-dgat2c*) or C-terminal (*dgat2c-egfp*). SE levels in WT, *dgat2c* KO, *dgat2c* KO/*dgat2c* OE, *dgat2c* KO/*egfp-dgat2c* OE, and *dgat2c* KO/*dgat2c-egfp* OE strains were measured using LC-ESI MS/MS (MRM).

**Fig. S7. Prediction of transmembrane regions of DGAT2C**

(A) Transmembrane regions of DGAT2C were predicted by Deep TMHMM (https://dtu.biolib.com/DeepTMHMM). (B) The amino acid sequence of DGAT2C. Predicted transmembrane (TM) regions of DGAT2C are indicated by magenta.

**Fig. S8. Synthetic activity of Δ7-stigmasterol ester of the putative transmembrane regions-deleted DGAT2C**

Chromatogram of C22:6-Δ7-stigmasterol ester (C22:6-Δ7-SE) of each strain detected using LC-ESI MS/MS analysis. A series of N-terminal-truncated mutants of DGAT2C were reintroduced into the *dgat2c* KO strain, and their C22:6-Δ7-SE levels were measured using LC-ESI MS/MS (MRM mode).

**Fig. S9. Heterologous expression of transmembrane-deleted DGAT2C and verification of regions contributing to the increase in SE synthesis**

(A) EE levels in the full-length, ΔTM1-6, ΔTM1-8, and ΔTM1-10 *dgat2c* expressing *S. cerevisiae*. The peak intensity of each EE was normalized with that of PC32:0. (B) Western blotting showing the protein expression level of ΔTM1-6 and ΔTM1-8 DGAT2C in *A. limacinum*. (C) Quantification of C22:6-CE of ΔTM1-6 and ΔTM1-8 DGAT2C expressing *A. limacinum*. Data represent means ± SD of three separate experiments. The peak intensity of each lipid was normalized with that of the internal standard.

**Fig. S10. Relative quantification of a free form of sterol in *A. limacinum***

(A) Schematic of methanolysis generating the free form of sterol from SE. (B) The relative amount of the free form of cholesterol of the WT, *dgat2c* KO, *dgat2c* KO/full-length *dgat2c* OE, and *dgat2c* KO/ΔTM1-8 *dgat2c* OE, and *dgat2c* KO/ΔTM1-10 *dgat2c* OE strains. The free form of cholesterol was measured using LC-APCI MS/MS (MRM mode) without methanolysis. The relative amount with the WT as 100% of cholesterol is calculated. Data represent means ± SD of three separate experiments.

**Fig. S11. Structural analysis of Δ7-cholesterol ester generated by ΔTM1-8 DGAT2C**

(A) Positive ion MS spectra of SE generated in the *dgat2c* KO/ΔTM1-8 *dgat2c* OE strain. The spectrum showing *m/z* 712.5 was rarely detected in the WT, while it was detected in the *dgat2c* KO/ΔTM1-8 *dgat2c* OE strain. (B) MS/MS fragmentation spectra of *m/z* 712.5. Structure-specific fragment ions are generated in MS/MS depending on the number of the double bond in ring B of the sterol moieties of SE. According to their MS/MS fragmentation pattern, *m/z* 712.5 was identified as a C22:6-Δ7-cholesterol ester.

**Fig. S12. Prediction of three-dimensional structures of DGAT2D and DGAT2C**

The three-dimensional structures of DGAT2D (A) and DGAT2C (B) are predicted by AlphaFold (https://alphafold.ebi.ac.uk/). A per-residue confidence score (pLDDT) between 0 and 100 is evaluated in AlphaFold. Model confidence is as follows: blue, very high (pLDDT>90); light blue, confident (90>pLDDT>70); yellow, low (70>pLDDT>50); orange, very low (pLDDT<50).

**Fig. S13. Multiple alignment of amino acid sequences of DGAT2C of thraustochytrids**

Multiple alignment of the amino acid sequence of DGAT2C of five genera of thraustochytrids: *Aurantiochytrium limacinum, Aplanochytrium kerguelense, Schizochytrium aggregatum, Thraustochytrium aureum*, and *Parietichytrium sarkarianum*.

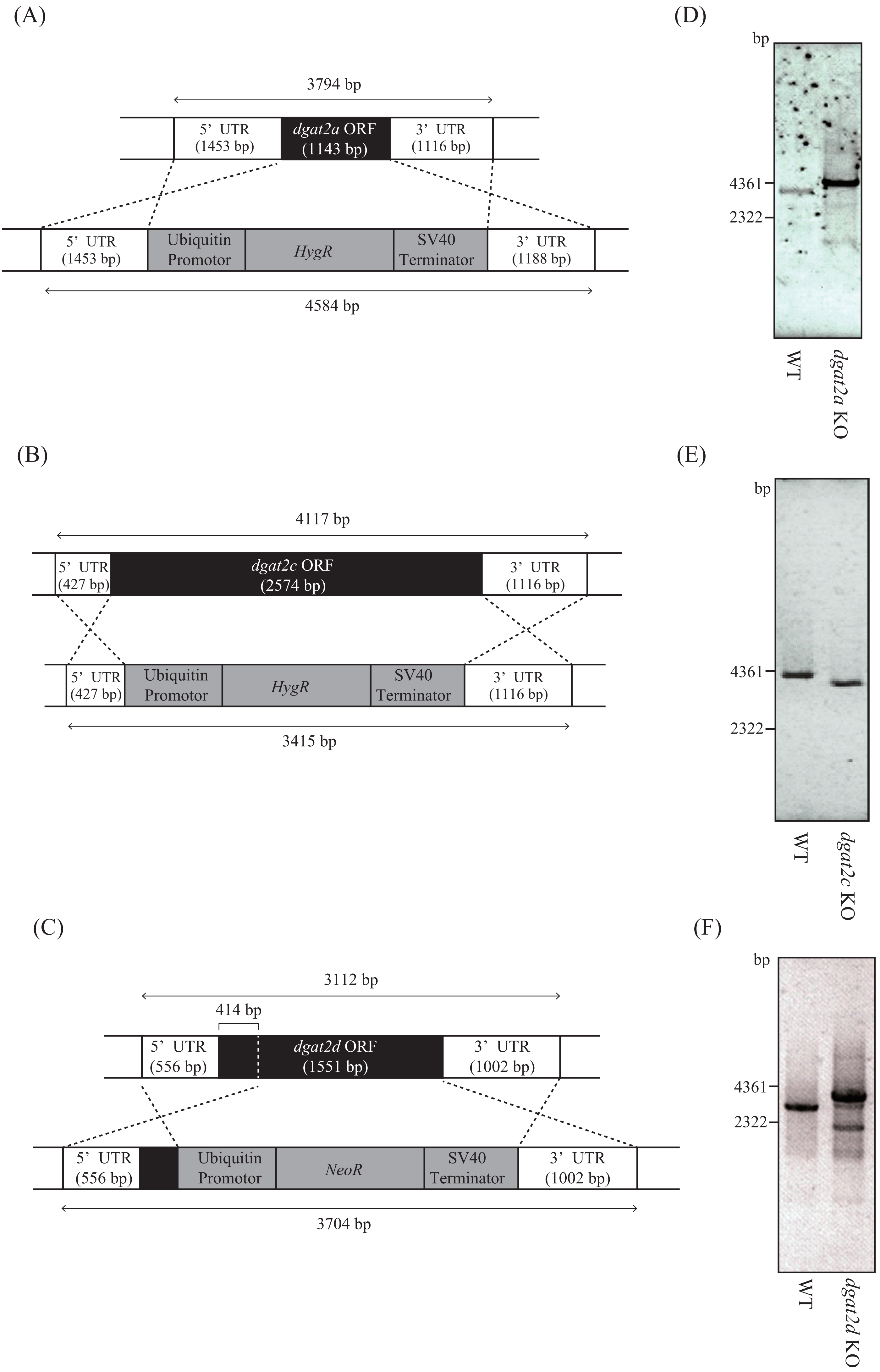
(A)
3794 bp
5' UTR (1453 bp)
dgat2a ORF (1143 bp)
3' UTR (1116 bp)
5' UTR (1453 bp)
Ubiquitin Promotor
HygR
SV40 Terminator
3' UTR (1188 bp)
4584 bp
(B)
4117 bp
5' UTR (427 bp)
dgat2c ORF (2574 bp)
3' UTR (1116 bp)
5' UTR (427 bp)
Ubiquitin Promotor
HygR
SV40 Terminator
3' UTR (1116 bp)
3415 bp
(C)
3112 bp
414 bp
5' UTR (556 bp)
dgat2d ORF (1551 bp)
3' UTR (1002 bp)
5' UTR (556 bp)
Ubiquitin Promotor
NeoR
SV40 Terminator
3' UTR (1002 bp)
3704 bp
(D)
bp
4361
2322
WT
dgat2a KO
(E)
bp
4361
2322
WT
dgat2c KO
(F)
bp
4361
2322
WT
dgat2d KO

Figure S1

(A)

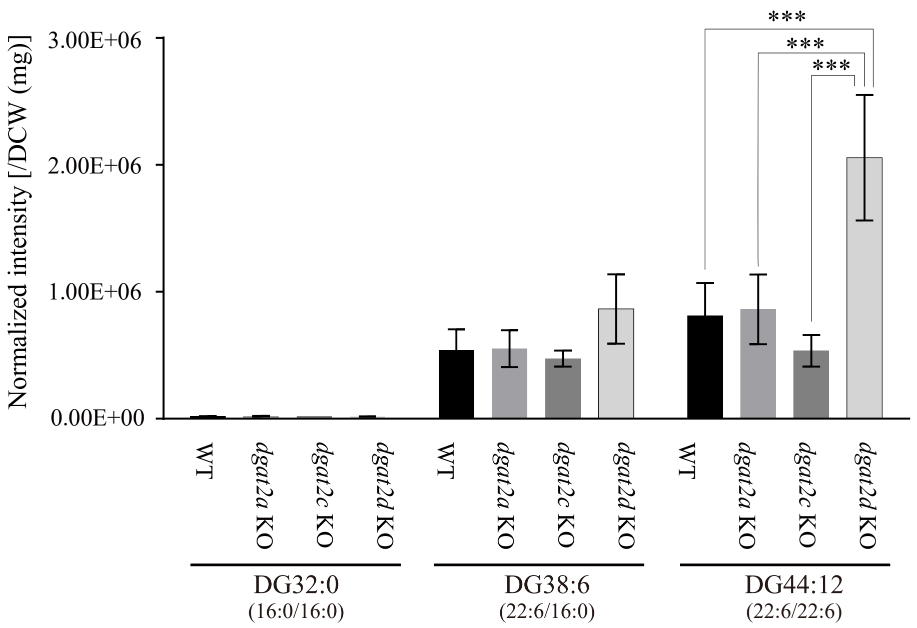
Normalized intensity [/DCW (mg)]
3.00E+06
2.00E+06
1.00E+06
0.00E+00
***
***
***
WT
dgat2a KO
dgat2c KO
dgat2d KO
DG32:0
(16:0/16:0)
DG38:6
(22:6/16:0)
DG44:12
(22:6/22:6)

(B)

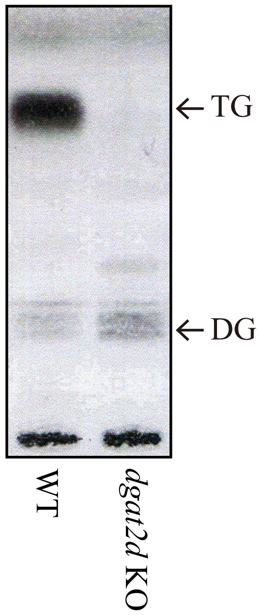
TG
DG
WT
dgat2d KO

Figure S2

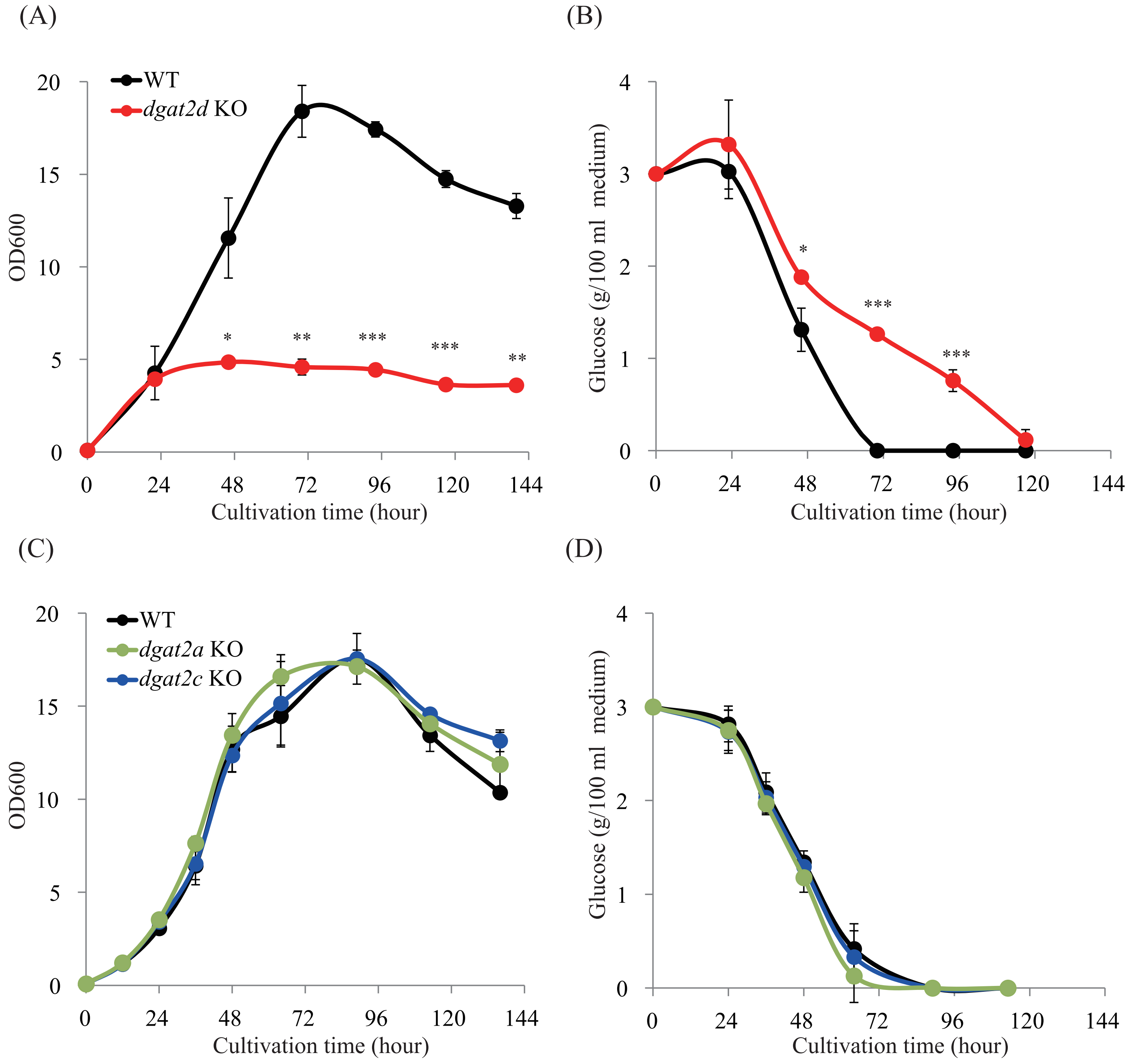

(A)
WT
dgat2d KO
OD600
Cultivation time (hour)
*
**
***
***
**
(B)
Glucose (g/100 ml medium)
Cultivation time (hour)
*
***
***
(C)
WT
dgat2a KO
dgat2c KO
OD600
Cultivation time (hour)
(D)
Glucose (g/100 ml medium)
Cultivation time (hour)


Figure S3

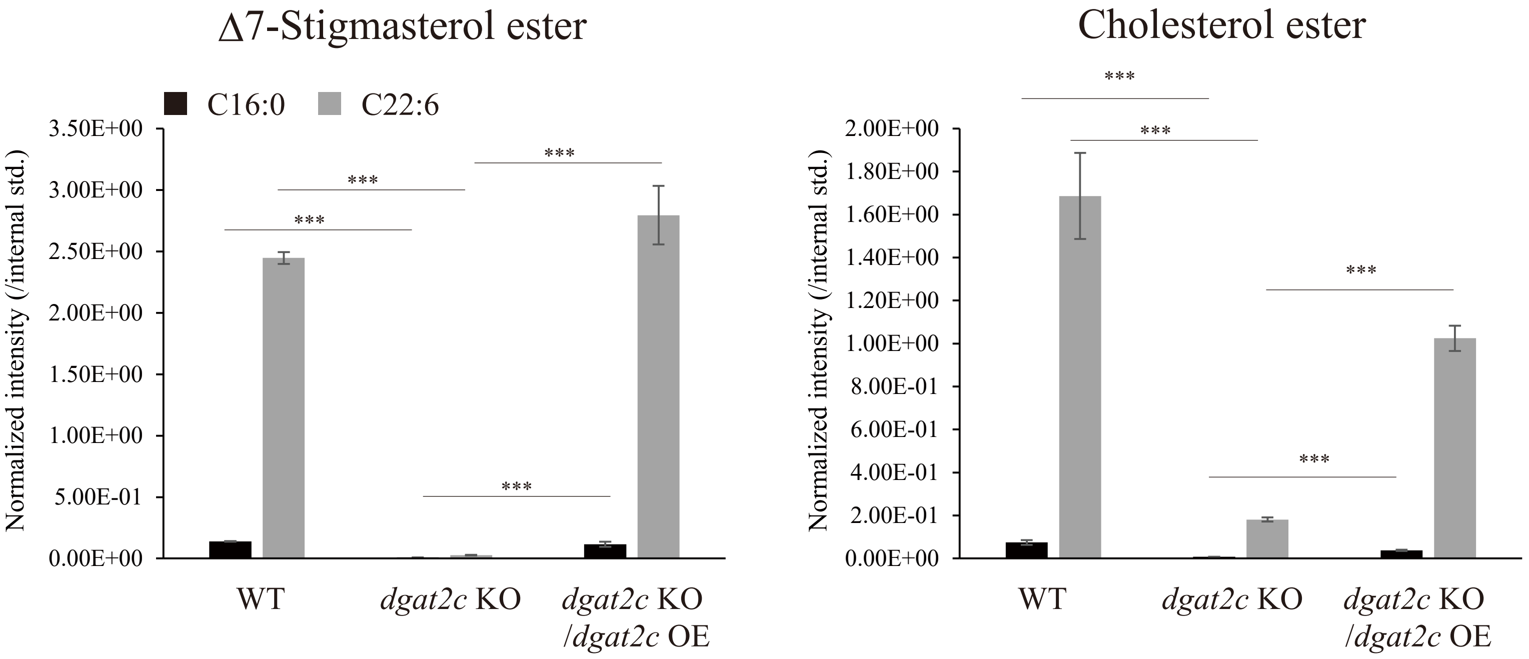

Δ7-Stigmasterol ester
C16:0
C22:6
Normalized intensity (/internal std.)
3.50E+00
3.00E+00
2.50E+00
2.00E+00
1.50E+00
1.00E+00
5.00E-01
0.00E+00
***
WT
dgat2c KO
dgat2c KO /dgat2c OE
Cholesterol ester
2.00E+00
1.80E+00
1.60E+00
1.40E+00
1.20E+00
1.00E+00
8.00E-01
6.00E-01
4.00E-01
2.00E-01
0.00E+00


Figure S4

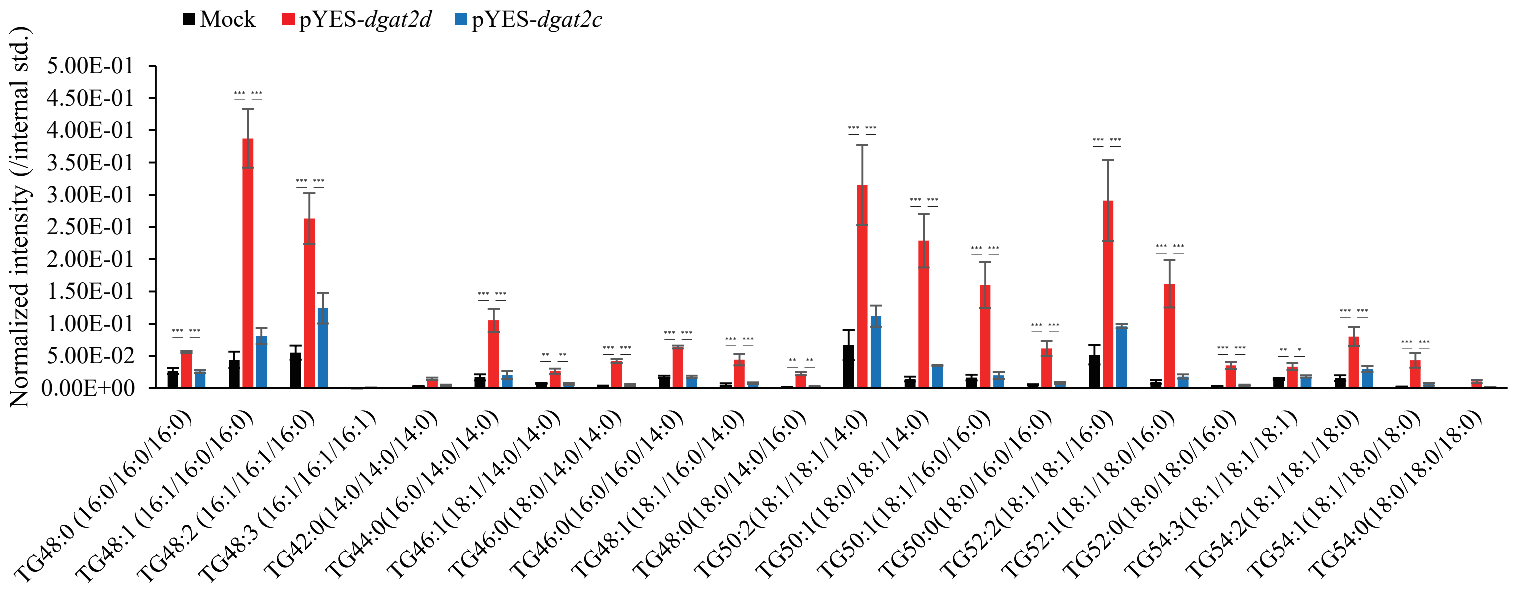

Mock
pYES-dgat2d
pYES-dgat2c
Normalized intensity (/internal std.)
5.00E-01
4.50E-01
4.00E-01
3.50E-01
3.00E-01
2.50E-01
2.00E-01
1.50E-01
1.00E-01
5.00E-02
0.00E+00
TG48:0 (16:0/16:0/16:0)
TG48:1 (16:1/16:0/16:0)
TG48:2 (16:1/16:1/16:0)
TG48:3 (16:1/16:1/16:1)
TG42:0(14:0/14:0/14:0)
TG44:0(16:0/14:0/14:0)
TG46:1(18:1/14:0/14:0)
TG46:0(18:0/14:0/14:0)
TG46:0(16:0/16:0/14:0)
TG48:1(18:1/16:0/14:0)
TG48:0(18:0/14:0/16:0)
TG50:2(18:1/18:1/14:0)
TG50:1(18:0/18:1/14:0)
TG50:1(18:1/16:0/16:0)
TG50:0(18:0/16:0/16:0)
TG52:2(18:1/18:1/16:0)
TG52:1(18:1/18:0/16:0)
TG52:0(18:0/18:0/16:0)
TG54:3(18:1/18:1/18:1)
TG54:2(18:1/18:1/18:0)
TG54:1(18:1/18:0/18:0)
TG54:0(18:0/18:0/18:0)


Figure S5

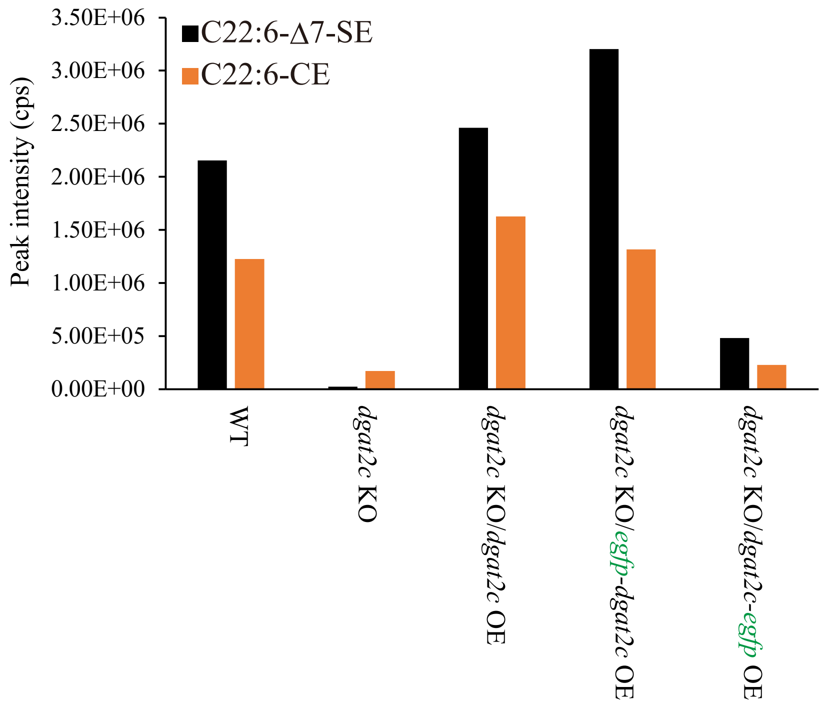

3.50E+06
3.00E+06
2.50E+06
2.00E+06
1.50E+06
1.00E+06
5.00E+05
0.00E+00
Peak intensity (cps)
C22:6-Δ7-SE
C22:6-CE
WT
dgat2c KO
dgat2c KO/dgat2c OE
dgat2c KO/egfp-dgat2c OE
dgat2c KO/dgat2c-egfp OE


Figure S6

(A)

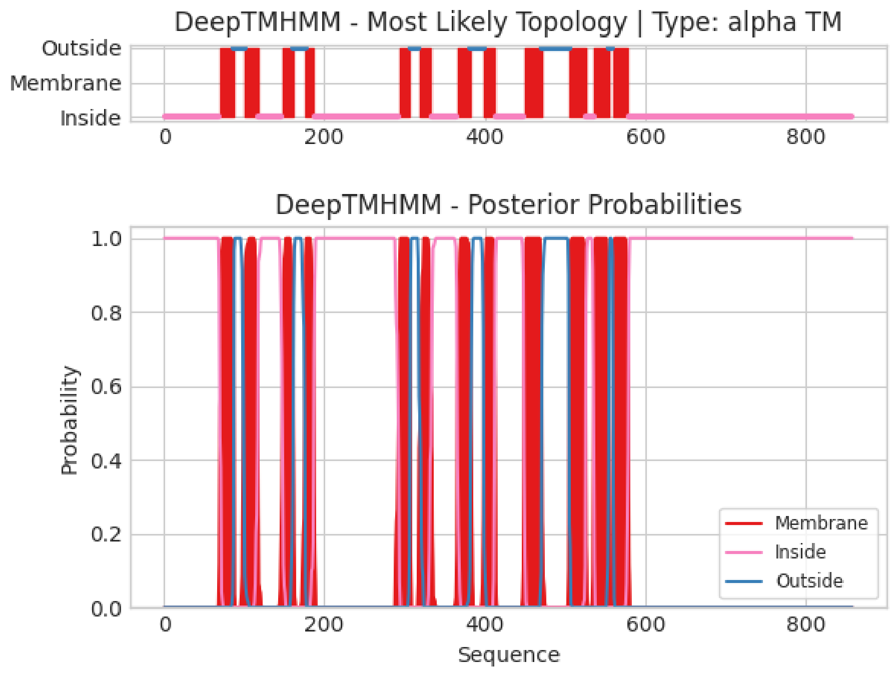


(B)

```
                                                                        TM1
        10         20         30         40         50         60         70         80
MAEGVEGDGK TQQARGQGTA SELATLKRDH EVLQNQFNEL QELYDTLLAE HEKQKSEKPQ QAFSAWLPYI KITVLWTLTA

                             TM2                                               TM3
        90        100        110        120        130        140        150        160
LVLFVVAERQ YTSGQKVGFG FLAACGGASL FLAIPATHWI GHLVHQDYKL FQPFRGGVRF VVLQAISWTF YGITAVIVMG

                        TM4
       170        180        190        200        210        220        230        240
GVAFAEHTNG GMLASAGVIG ILSQVFMVSS LLTYSNSSQS SRRARRIWRQ NSGVINDDDP ADELLEEERQ AYHAHRKSSA

                                                            TM5
       250        260        270        280        290        300        310        320
SDSEQSVDSE HPRRRAVVTS VIDRDMSDRM PFALEPETDK NIPSVVKEFV HMNTGLVVIG IALGLLSEHS PDIKYKPILG

   TM6                                               TM7
       330        340        350        360        370        380        390        400
LLSLMCCIVA ISLTHGIGGR LRHFHRGWSF AQSFRGGKEF VFLQAFGWCF FGISILAQGA FVISSAYLGK RSFKGTMYIG

   TM8                                                          TM9
       410        420        430        440        450        460        470        480
ALAAVVSHYA IVSSLPRFDQ KEDNKATDPR RYRVLESFTF WEHLSAWPIM ILFCNLQFMS FVVYMVLFTI PYFMPDEWQP

                                        TM10                 TM11
       490        500        510        520        530        540        550        560
WLAQNTLDLI PDLNAYESND FVHTFKLWWI LMWAVAFWTF FYSALNNWGI EGWRQSISLS LAAVAAYGGV LSAFRDSPHY

   TM12
       570        580        590        600        610        620        630        640
MMLLVICTAN LVYISTTFTK RPEFNACREW DLFRRLQFLP RIVEKYFGLR VELTEEMQKF APYLGDENAK DPRMHQVLLL

       650        660        670        680        690        700        710        720
FHPHGIFPVT HAVLPLSLTW RKLFPKLQVN SLSATINHII PVMRDVIQWM GVCDVSRATV MNLIRMGRNL QIVCGGQTEM

       730        740        750        760        770        780        790        800
FESRSWDKKI AIVRKRRRGI FKIAIQQGLG IVPMYSFGEP QIFDNVYMPR TQAFFKRLLG FPFPFFMIGK FGLPIPCRVP

       810        820        830        840        850        860        870        880
VTVAIDAPVF PVKQNSNPTP EEVTEFQYRY FEKLEALFER YKHDNGHGEH ELDFIDY
```

Figure S7

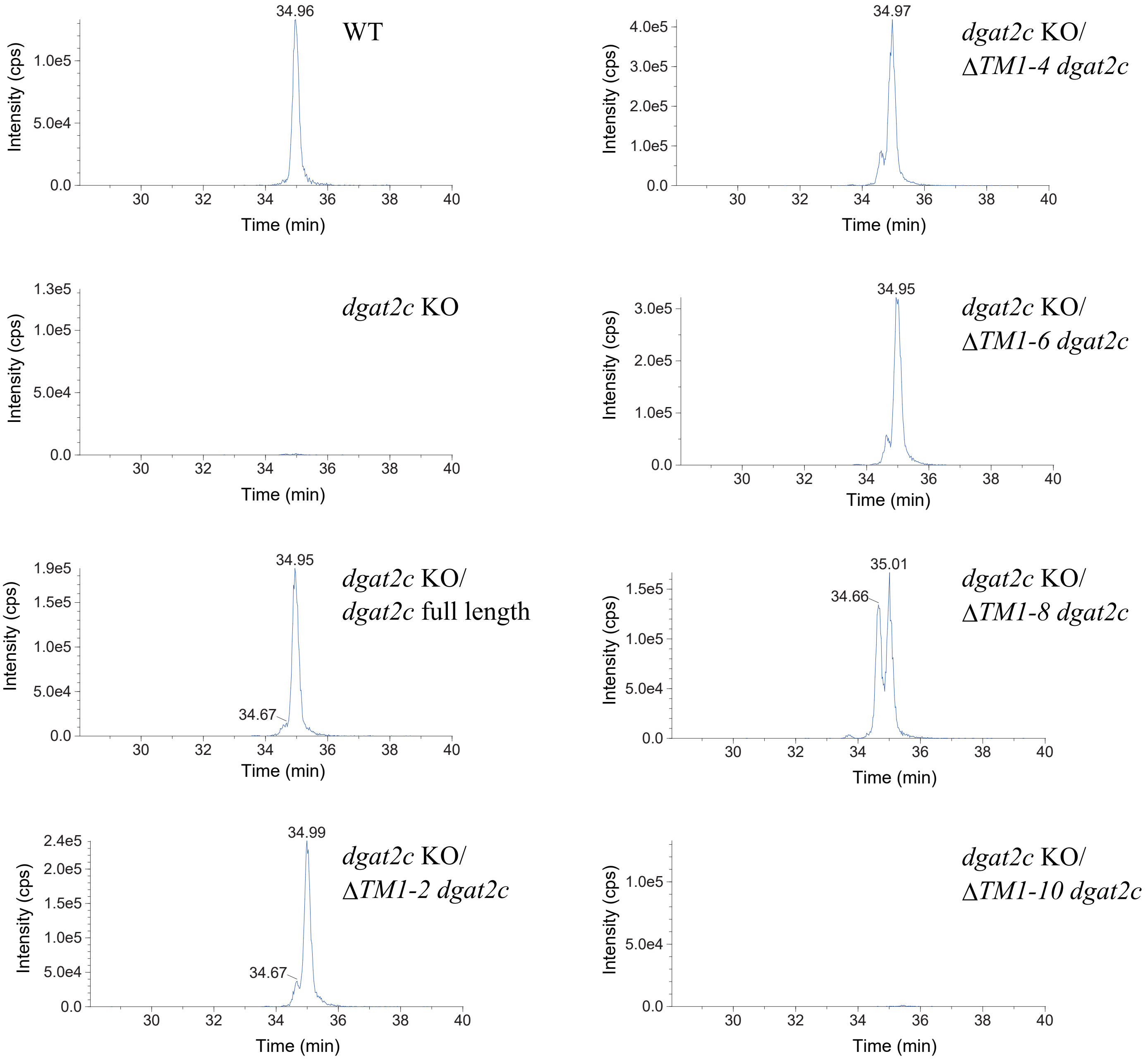
WT
34.96
dgat2c KO
dgat2c KO/
dgat2c full length
34.95
34.67
dgat2c KO/
ΔTM1-2 dgat2c
34.99
34.67
dgat2c KO/
ΔTM1-4 dgat2c
34.97
dgat2c KO/
ΔTM1-6 dgat2c
34.95
dgat2c KO/
ΔTM1-8 dgat2c
35.01
34.66
dgat2c KO/
ΔTM1-10 dgat2c
Intensity (cps)
Time (min)

Figure S8

(A)

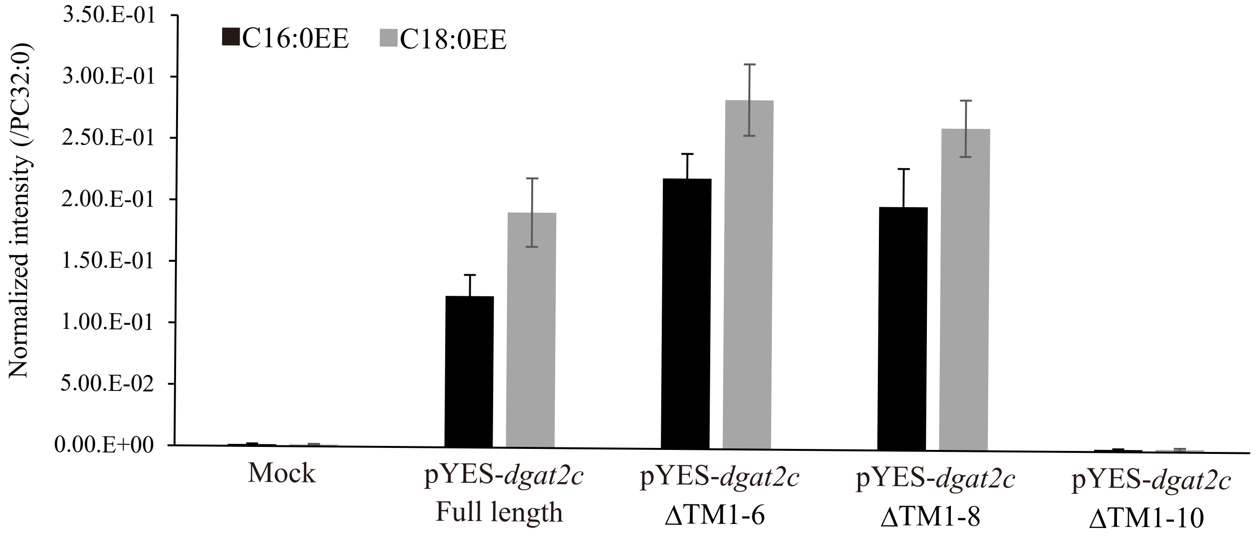


(B)

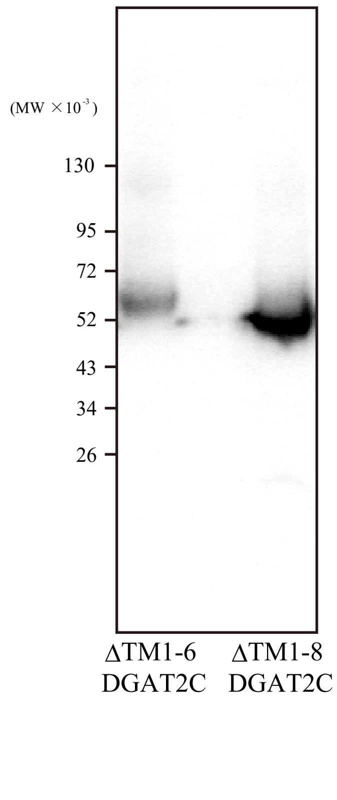


(C)

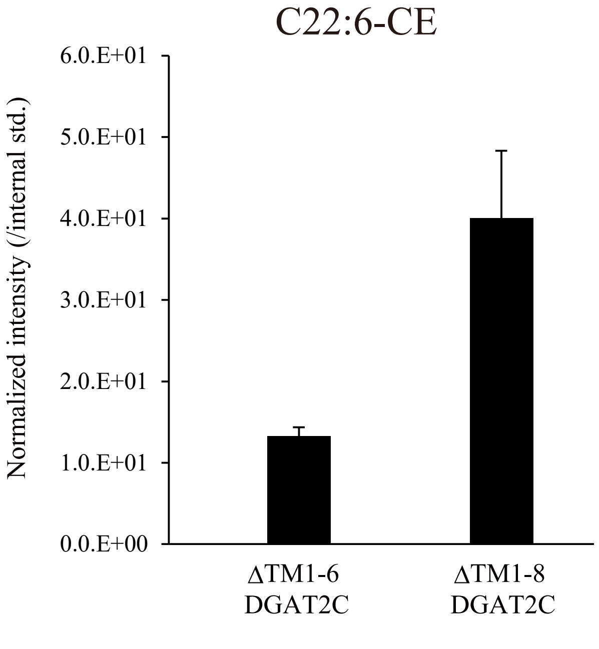


Figure S9

(A)

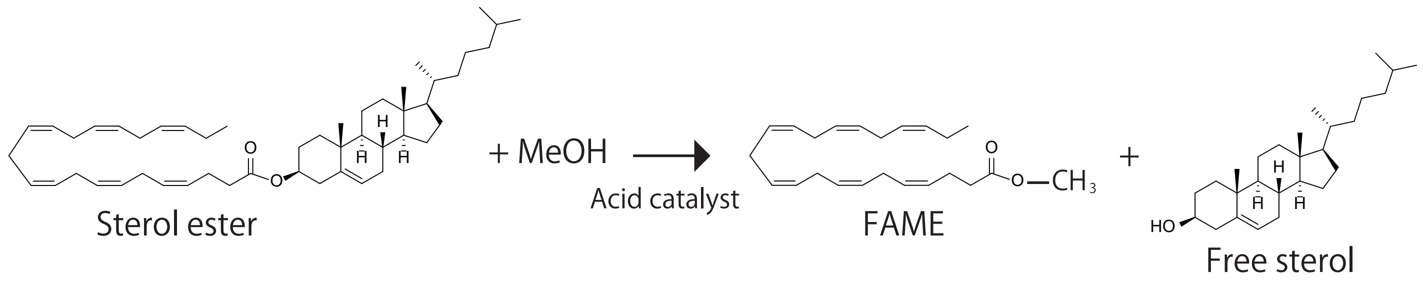


(B)

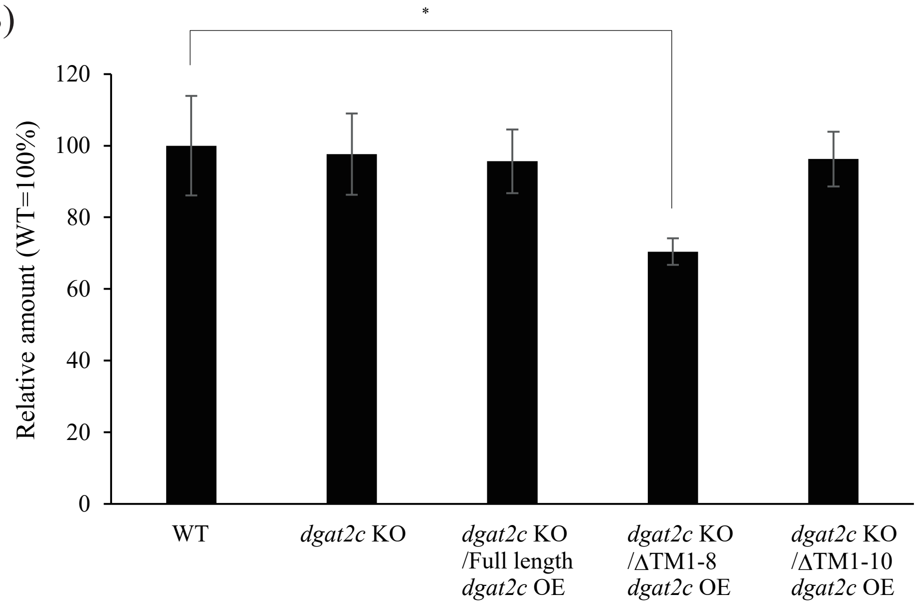


Figure S10

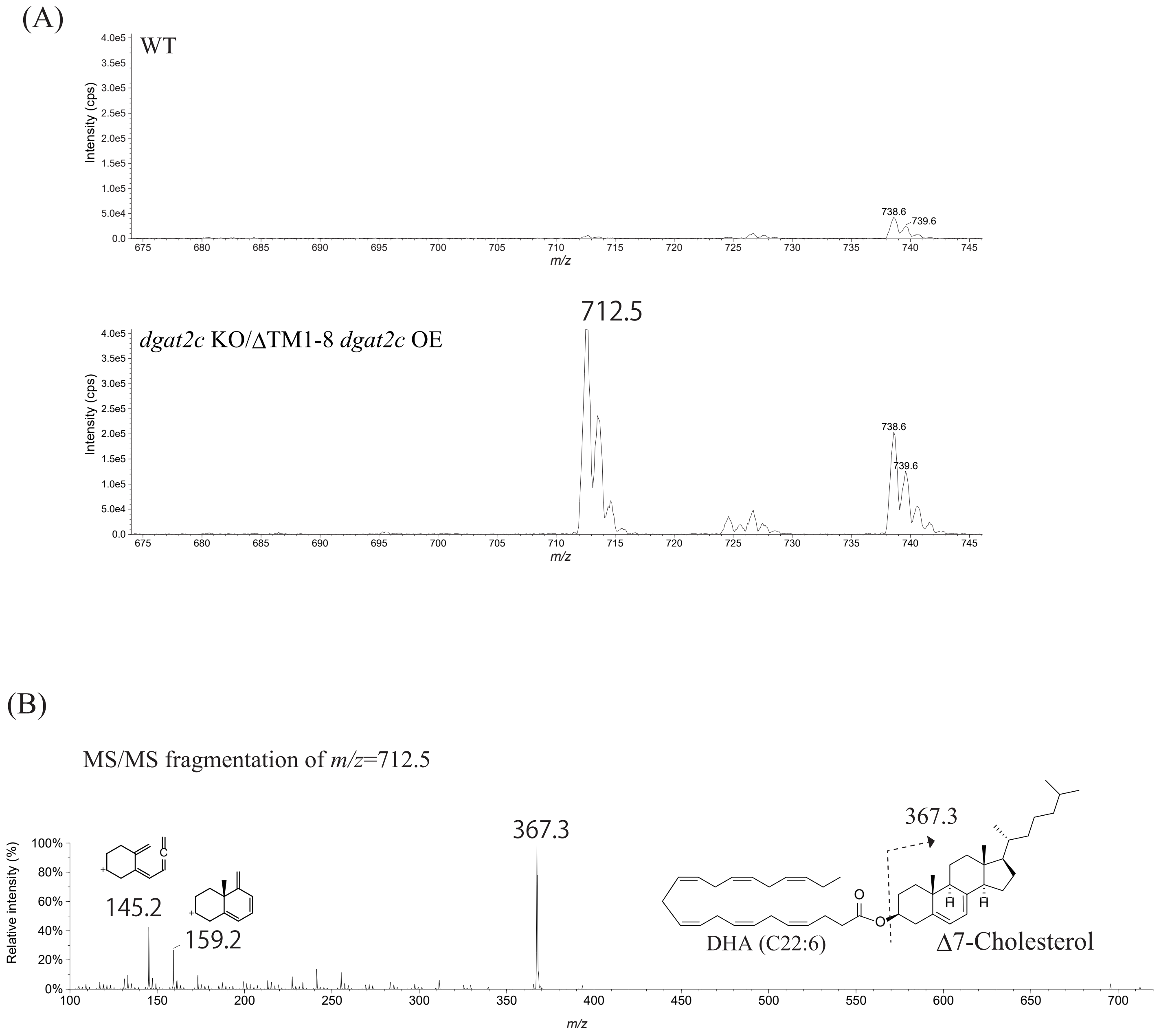

(A)
WT
Intensity (cps)
738.6
739.6
m/z
712.5
dgat2c KO/ΔTM1-8 dgat2c OE
738.6
739.6
(B)
MS/MS fragmentation of m/z=712.5
367.3
145.2
159.2
Relative intensity (%)
DHA (C22:6)
Δ7-Cholesterol
m/z


Figure S11

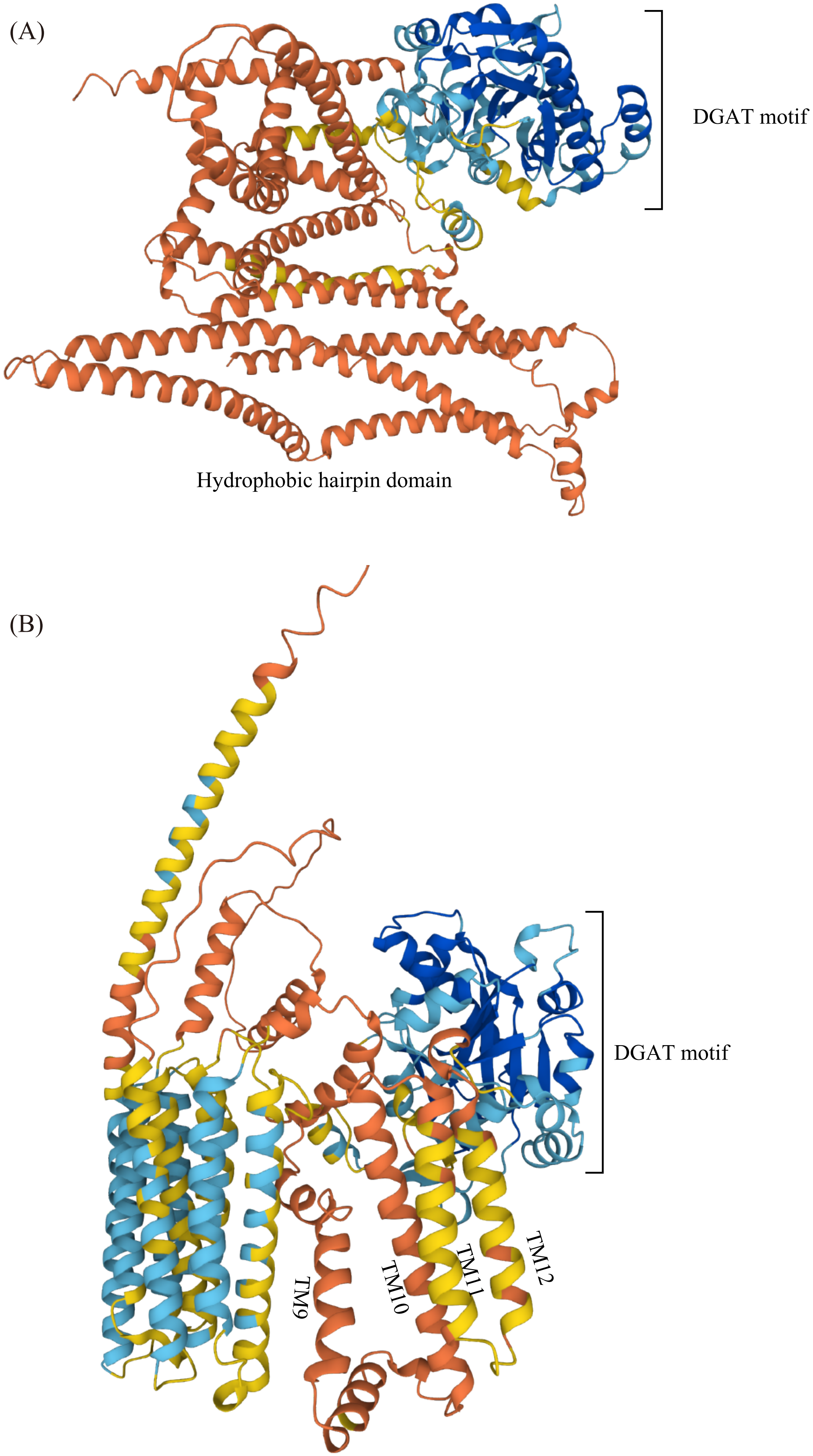
(A)
DGAT motif
Hydrophobic hairpin domain
(B)
DGAT motif
TM9
TM10
TM11
TM12

Figure S12

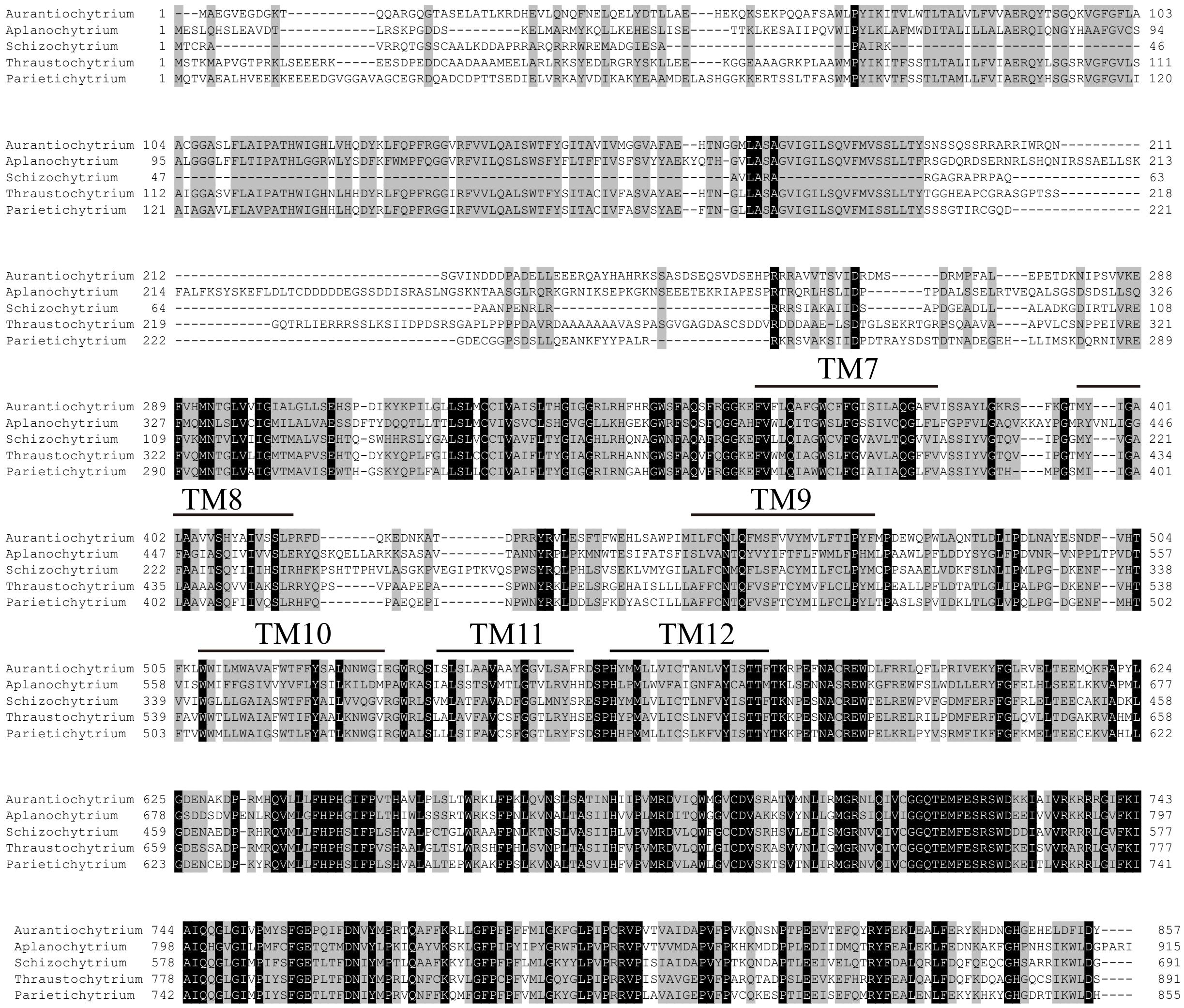


Figure S13
